# Supplementary material for: Allosteric modulation of Plasmodium falciparum Isoleucyl tRNA synthetase by South African natural compounds
Source: PLoS One. 2025 May 14;20(5):e0321444. doi: 10.1371/journal.pone.0321444 (PMC12077802; doi:10.1371/journal.pone.0321444)
Supplement: S1 File — PDB template structures validation report and PfIleRS and HsIleRS verify3D and ProSA validation reports. (A) 7D5C template resolution of 1.9 Å and Rfree of 0.20. (B) 6LDK Resolution of 2.9 Å and and Rfree of 0.24. (C) PfIleRS and HsIleRS scatter plots showing the percentage of residues that scored >= 0.1 in the 3D/1D profile. (D) The position of the modelled Plasmodium and human structures in references to experimentally determined structures (X-ray and Nuclear Magnetic Resonance (NMR). S2 Fig. Conservation scores of all predicted pockets generated from multiple sequence alignment of PfIleRS with other species of plasmodium: (Plasmodium malariae, Plasmodium knowlesi) and mammalian counterparts (Homo sapiens and Mus musculus). Normalised conservation scores are indicated for each residue in the alignment. S3 Fig. Ligplot analysis of modelled and redocked AMP complex interactions. (A) PfIleRS-AMP modelled and redocked residue interactions. (B) HsIleRS-AMP modelled and redocked residue interactions. The modelled AMP (grey) and redocked AMP (blue); while identical interacting residues are shown in red ellipses. S4 Fig. Protein-ligand interactions of selected potential SANCDB hit compounds against PfIleRS protein. All possible interactions formed between the PfIleRS receptor and the SANCDB compound hits are shown with their respective binding modes. S5 Fig. Binding modes and protein-ligand interactions of SANCDB potential hits in HsIleRS protein. S6 Fig. Global trajectory analysis of PfIleRS, PfIleRS orthosteric site and HsIleRS holo and ligand bound complexes. (A) Backbone-RMSD line plots of holo states and PfIleRS-SANC complexes. (B) Orthosteric pocket backbone-RMSD line plots for holo and PfIleRS-SANC systems. (C) Back-bone RMSD line plots for HsIleRS holo and ligand complexes. S7 Fig. PfIleRS-SANDB complexes superimposed trajectory snap shots of the 3 most sampled conformations. Dotted circles indicate the regions of PfIleRS structure where significant RMSD deviation [file pone.0321444.s001.pdf]

Supporting information

Allosteric Modulation of *Plasmodium falciparum* Isoleucyl tRNA Synthetase by South African Natural Compounds

Curtis Chepsiror[1], Wayde Veldman[1], Fisayo Olotu[1] and Özlem Tastan Bishop[1]*

[1]Research Unit in Bioinformatics (RUBi),
Department of Biochemistry, Microbiology and Bioinformatics,
Rhodes University, Makhanda, 6139, South Africa.

*Corresponding author:

Email: o.tastanbishop@ru.ac.za (OTB)

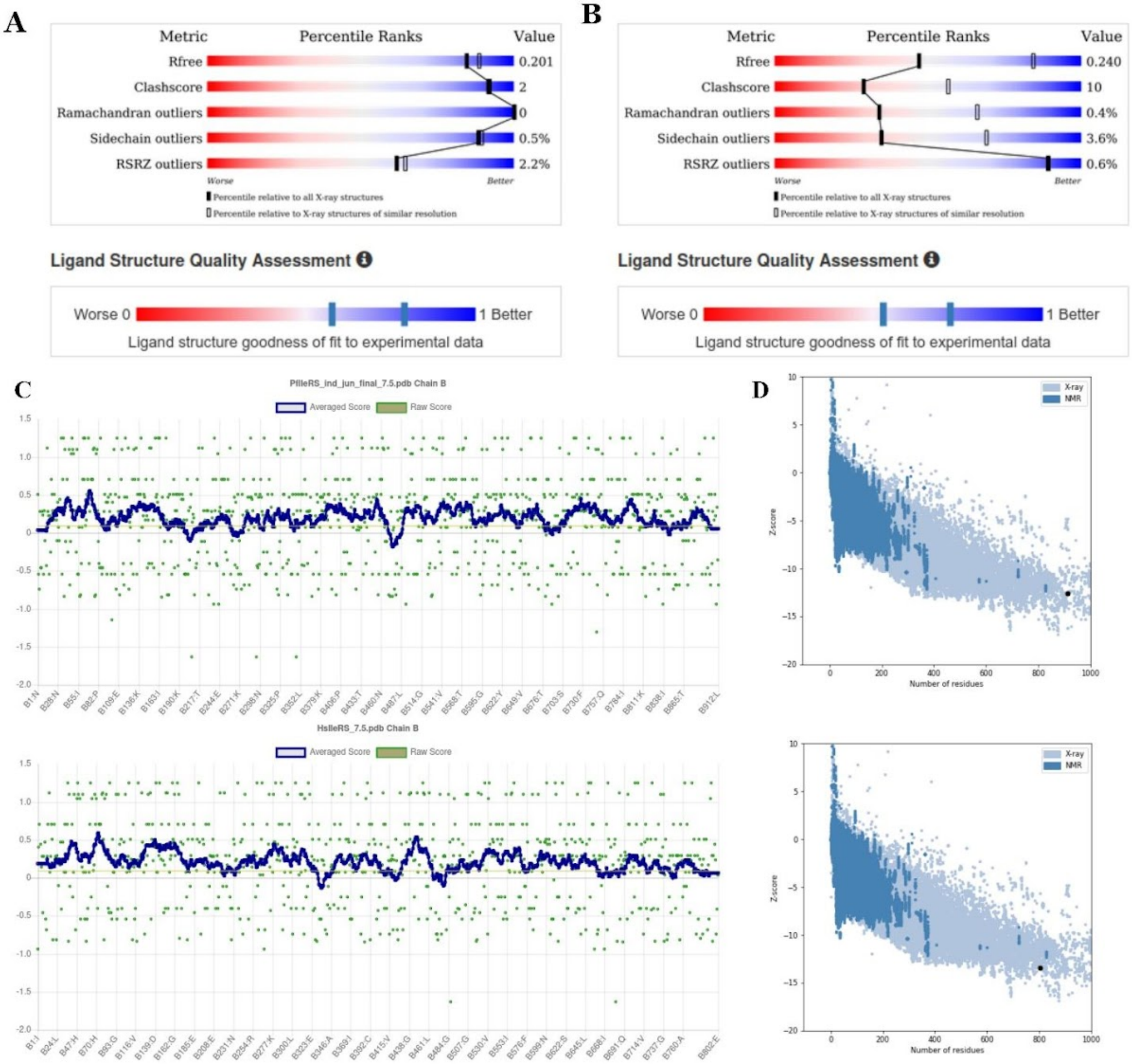
A
Metric
Percentile Ranks
Value
Rfree
0.201
Clashscore
2
Ramachandran outliers
0
Sidechain outliers
0.5%
RSRZ outliers
2.2%
Worse
Better
Percentile relative to all X-ray structures
Percentile relative to X-ray structures of similar resolution
Ligand Structure Quality Assessment
Worse 0
1 Better
Ligand structure goodness of fit to experimental data
B
Metric
Percentile Ranks
Value
Rfree
0.240
Clashscore
10
Ramachandran outliers
0.4%
Sidechain outliers
3.6%
RSRZ outliers
0.6%
Worse
Better
Percentile relative to all X-ray structures
Percentile relative to X-ray structures of similar resolution
Ligand Structure Quality Assessment
Worse 0
1 Better
Ligand structure goodness of fit to experimental data
C
PfIleRS_ind_jun_final_7.5.pdb Chain B
Averaged Score
Raw Score
HsIleRS_7.5.pdb Chain B
Averaged Score
Raw Score
D
X-ray
NMR
Z-score
Number of residues
X-ray
NMR
Z-score
Number of residues

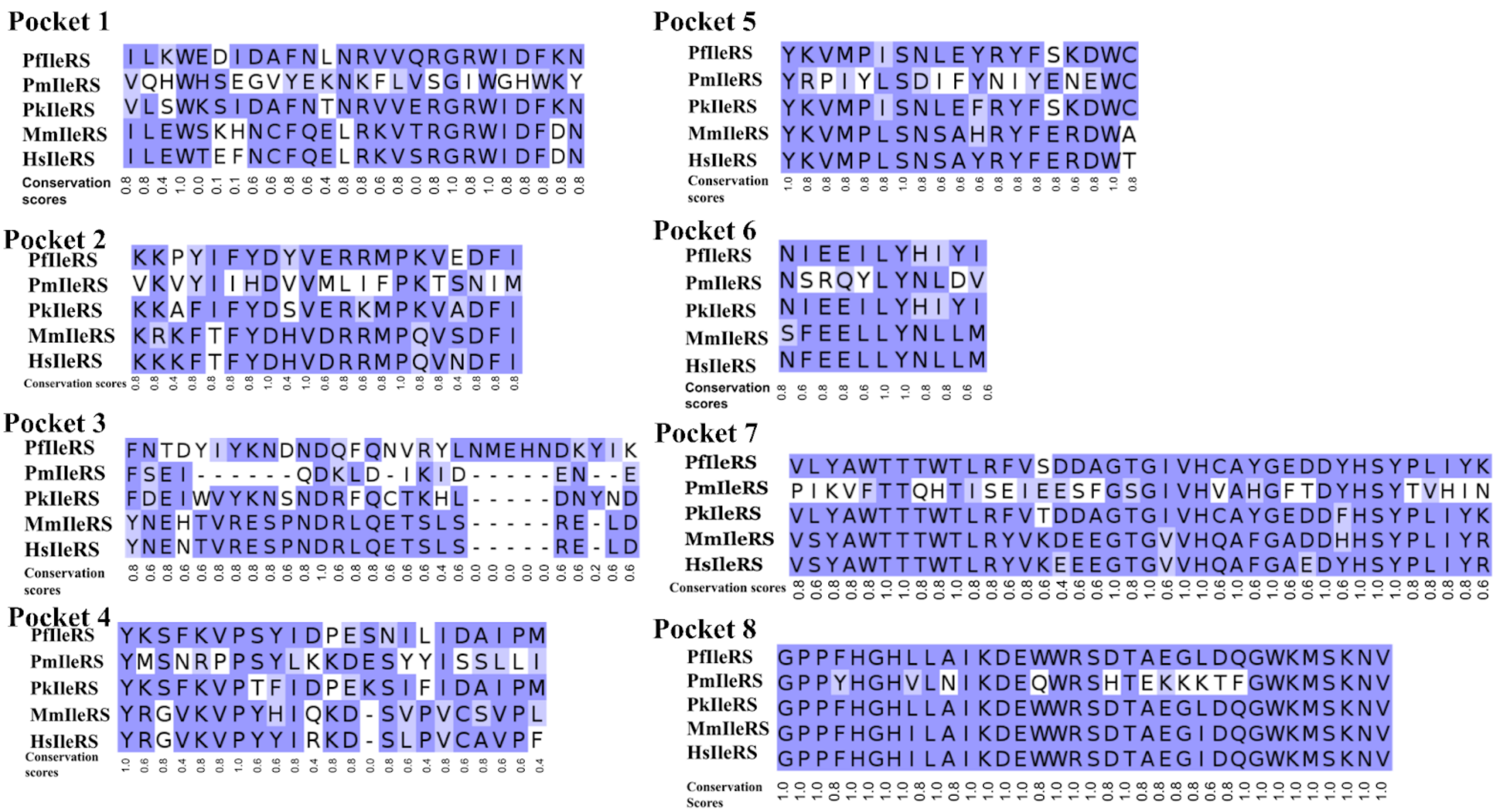
Pocket 1
PfIleRS ILKWEDIDAFNLNRVVQRGRWIDFKN
PmIleRS VQHWHSEGVYEKNKFLVSGIWGHWKY
PkIleRS VLSWKSIDAFNTNRVVERGRWIDFKN
MmIleRS ILEWSKHNCFQELRKVTRGRWIDFDN
HsIleRS ILEWTEFNCFQELRKVSRGRWIDFDN
Conservation scores 0.8 0.8 0.4 1.0 0.0 0.1 0.1 0.6 0.6 0.8 0.6 0.4 0.8 0.6 0.8 0.0 0.8 1.0 0.8 1.0 0.8 0.8 0.8 0.8 0.8 0.8
Pocket 2
PfIleRS KKPYIFYDYVERRMPKVEDFI
PmIleRS VKVYIIHDVVMLIFPKTSNIM
PkIleRS KKAFIFYDSVERKMPKVADFI
MmIleRS KRKFTFYDHVDRRMPQVSDFI
HsIleRS KKKFTFYDHVDRRMPQVNDFI
Conservation scores 0.8 0.8 0.4 0.8 0.8 0.8 0.8 1.0 0.4 1.0 0.6 0.8 0.6 0.8 1.0 0.8 0.8 0.4 0.8 0.8 0.8
Pocket 3
PfIleRS FNTDYIYKNDNDQFQNVRYLNMEHNDKYIK
PmIleRS FSEI------QDKLD-IKID-----EN--E
PkIleRS FDEIWVYKNSNDRFQCTKHL-----DNYND
MmIleRS YNEHTVRESPNDRLQETSLS-----RE-LD
HsIleRS YNENTVRESPNDRLQETSLS-----RE-LD
Conservation scores 0.8 0.6 0.8 0.6 0.6 0.8 0.8 0.8 0.8 0.6 0.8 1.0 0.6 0.8 0.8 0.6 0.6 0.6 0.4 0.6 0.0 0.0 0.0 0.0 0.0 0.6 0.6 0.2 0.6 0.6
Pocket 4
PfIleRS YKSFKVPSYIDPESNILIDAIPM
PmIleRS YMSNRPPSYLKKDESYYISSLLI
PkIleRS YKSFKVPTFIDPEKSIFIDAIPM
MmIleRS YRGVKVPYHIQKD-SVPVCSVPL
HsIleRS YRGVKVPYYIRKD-SLPVCAVPF
Conservation scores 1.0 0.6 0.8 0.4 0.8 0.8 1.0 0.6 0.6 0.8 0.4 0.8 0.8 0.0 0.8 0.6 0.4 0.8 0.6 0.8 0.6 0.6 0.4
Pocket 5
PfIleRS YKVMPISNLEYRYFSKDWC
PmIleRS YRPIYLSDIFYNIYENEWC
PkIleRS YKVMPISNLEFRYFSKDWC
MmIleRS YKVMPLSNSAHRYFERDWA
HsIleRS YKVMPLSNSAYRYFERDWT
Conservation scores 1.0 0.8 0.8 0.8 0.8 0.8 1.0 0.8 0.6 0.6 0.6 0.8 0.8 0.8 0.8 0.6 0.8 1.0 0.8
Pocket 6
PfIleRS NIEEILYHIYI
PmIleRS NSRQYLYNLDV
PkIleRS NIEEILYHIYI
MmIleRS SFEELLYNLLM
HsIleRS NFEELLYNLLM
Conservation scores 0.8 0.6 0.8 0.8 0.6 1.0 1.0 0.8 0.8 0.6 0.6
Pocket 7
PfIleRS VLYAWTTTWTLRFVSDDAGTGIVHCAYGEDDYHSYPLIYK
PmIleRS PIKVFTTQHTISEIEESFGSGIVHVAHGFTDYHSYTVHIN
PkIleRS VLYAWTTTWTLRFVTDDAGTGIVHCAYGEDDFHSYPLIYK
MmIleRS VSYAWTTTWTLRYVKDEEGTGVVHQAFGADDHHSYPLIYR
HsIleRS VSYAWTTTWTLRYVKEEEGTGVVHQAFGAEDYHSYPLIYR
Conservation scores 0.8 0.6 0.8 0.8 0.8 1.0 1.0 0.8 0.8 1.0 0.8 0.8 0.6 0.8 0.6 0.4 0.6 0.6 1.0 0.8 1.0 0.6 1.0 1.0 0.6 1.0 0.6 1.0 0.6 0.6 1.0 0.6 1.0 1.0 1.0 0.8 0.8 0.8 0.8 0.6
Pocket 8
PfIleRS GPPFHGHLLAIKDEWWRSDTAEGLDQGWKMSKNV
PmIleRS GPPYHGHVLNIKDEQWRSHTEKKKTFGWKMSKNV
PkIleRS GPPFHGHLLAIKDEWWRSDTAEGLDQGWKMSKNV
MmIleRS GPPFHGHILAIKDEWWRSDTAEGIDQGWKMSKNV
HsIleRS GPPFHGHILAIKDEWWRSDTAEGIDQGWKMSKNV
Conservation Scores 1.0 1.0 1.0 0.8 1.0 1.0 1.0 1.0 0.8 1.0 0.8 1.0 1.0 1.0 1.0 0.8 1.0 1.0 1.0 0.8 1.0 0.8 0.8 0.8 0.8 0.6 0.8 1.0 1.0 1.0 1.0 1.0 1.0 1.0 1.0 1.0

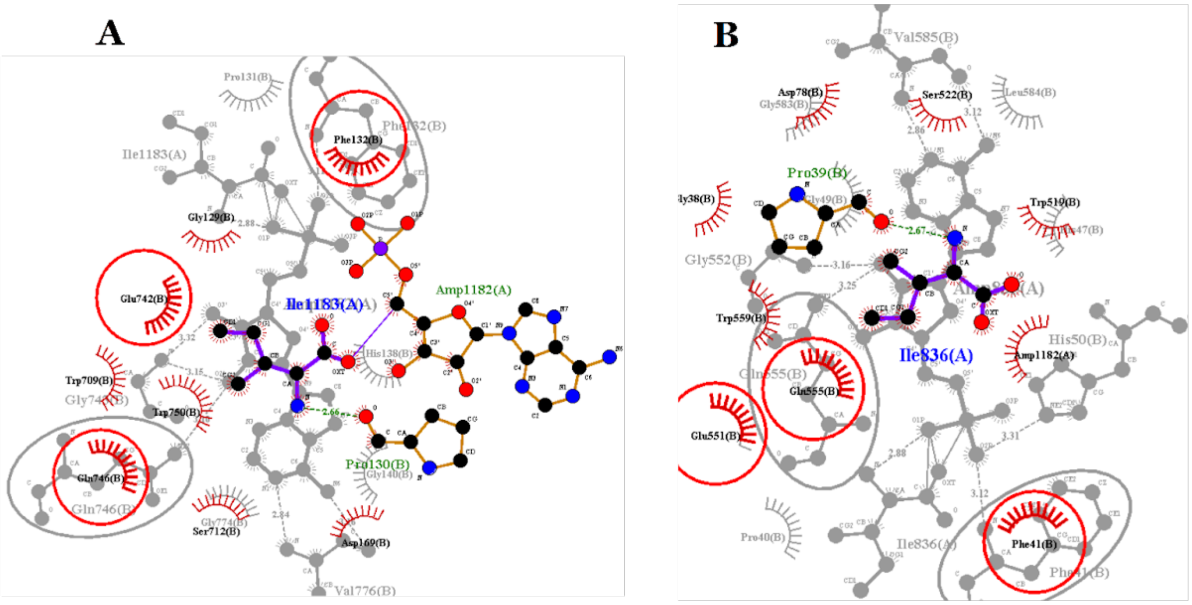

A
Ile1183(A)
Amp1182(A)
Pro130(B)
Phe132(B)
Glu742(B)
Gly129(B)
Trp709(B)
Trp750(B)
Gln746(B)
Ser712(B)
Asp169(B)
Val776(B)
Pro131(B)
B
Ile836(A)
Pro39(B)
Val585(B)
Asp78(B)
Ser522(B)
Leu584(B)
Trp519(B)
Gly552(B)
Trp559(B)
Gln555(B)
Glu551(B)
His50(B)
Amp1182(A)
Pro40(B)
Phe41(B)

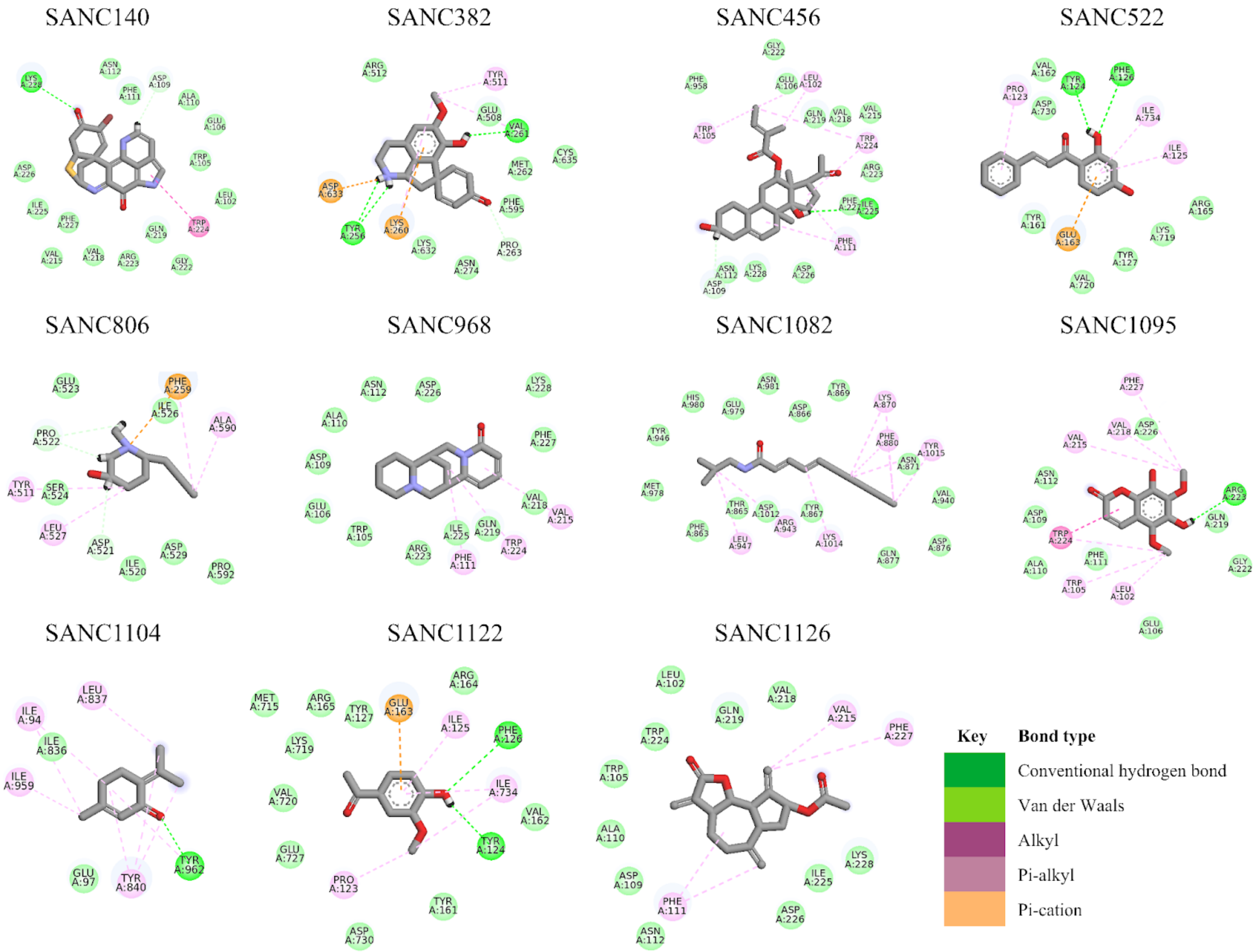
SANC140
SANC382
SANC456
SANC522
SANC806
SANC968
SANC1082
SANC1095
SANC1104
SANC1122
SANC1126
Key
Bond type
Conventional hydrogen bond
Van der Waals
Alkyl
Pi-alkyl
Pi-cation

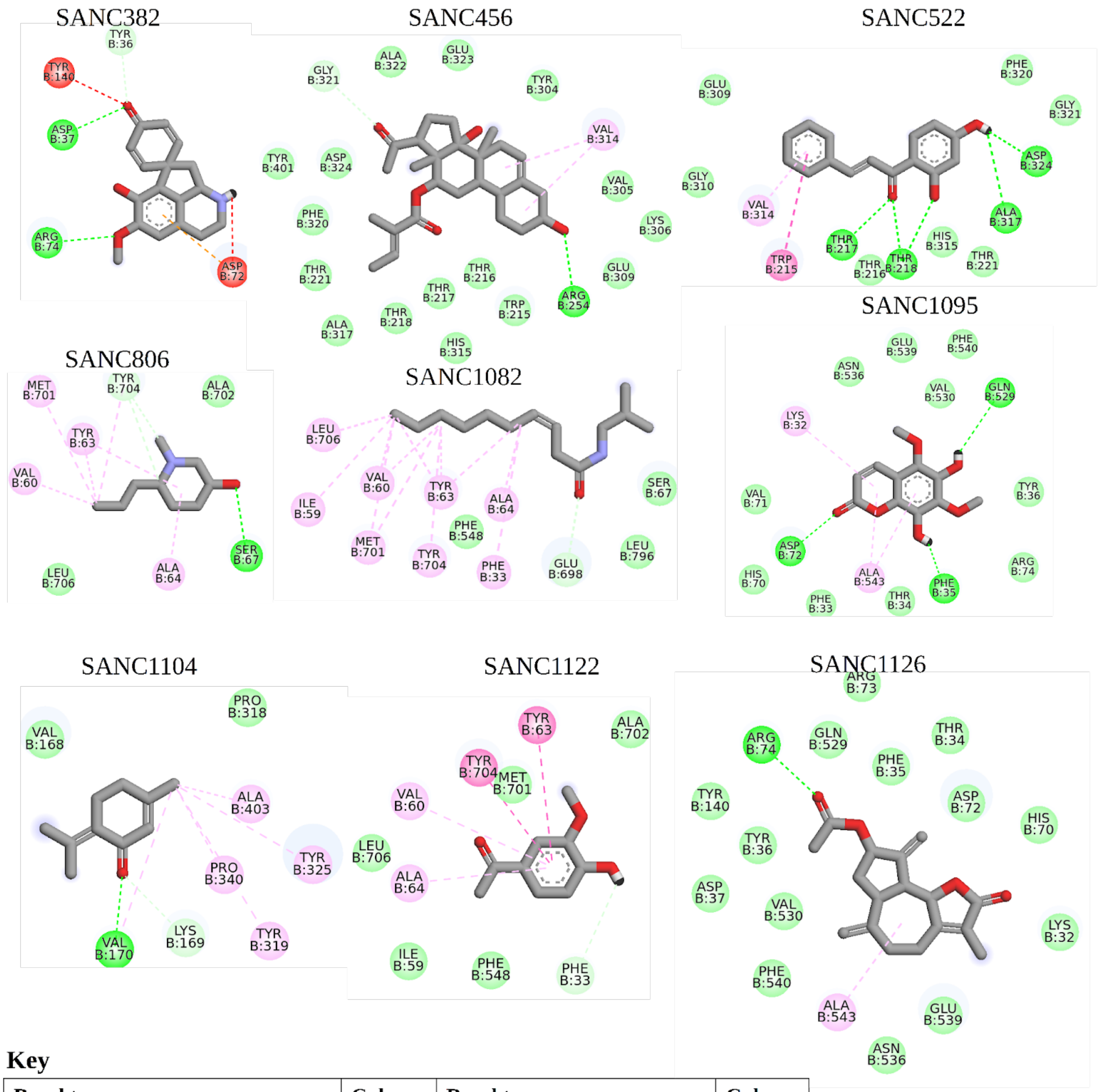

Key

Bond type	Colour	Bond type	Colour
Conventional hydrogen bond		Alkyl bond	
Carbon hydrogen bond		Pi-alkyl bond	
Van der Waals		Pi-Anion	
Unfavorable acceptor-acceptor		Unfavorable donor-donor	

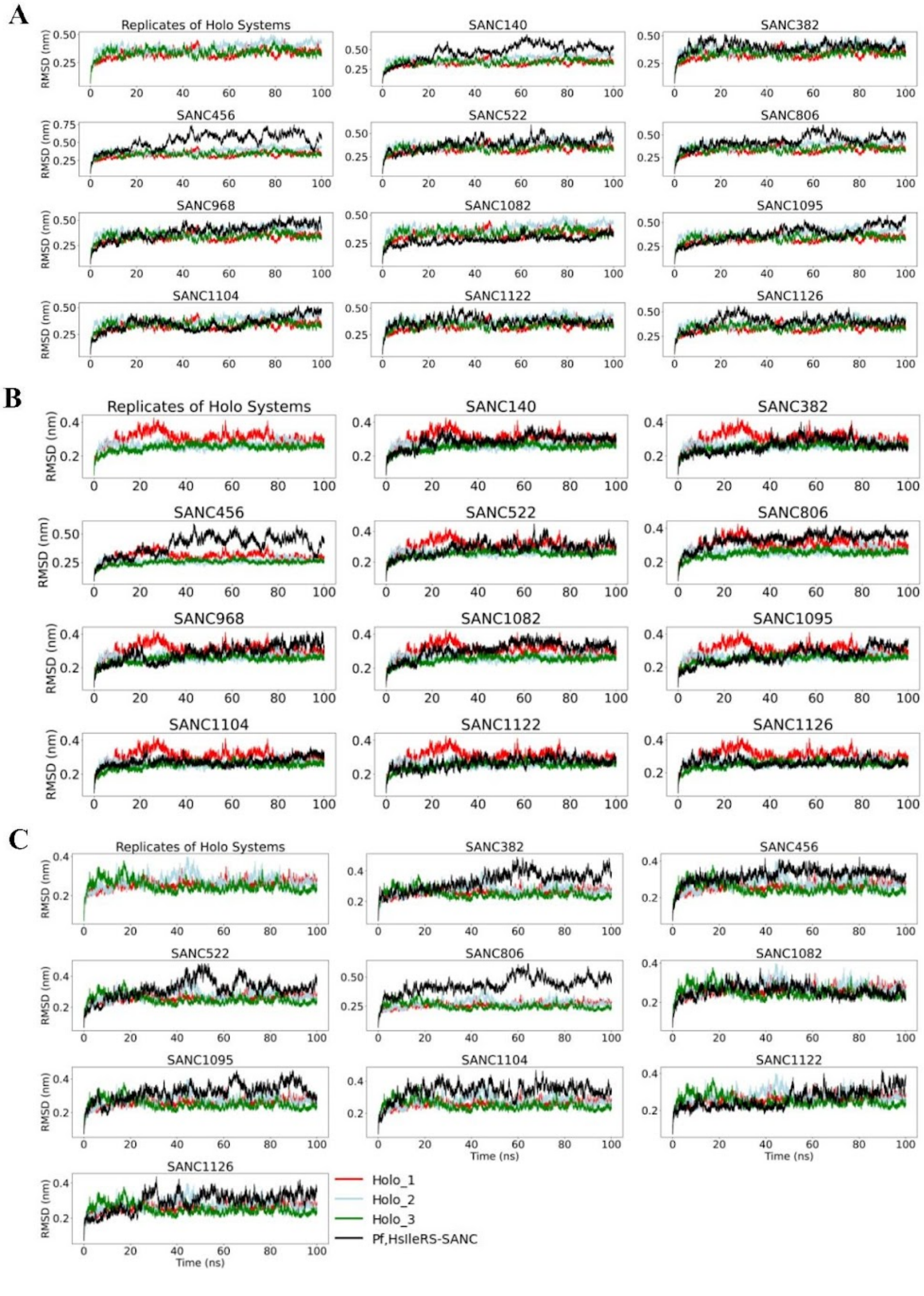
A
Replicates of Holo Systems
SANC140
SANC382
SANC456
SANC522
SANC806
SANC968
SANC1082
SANC1095
SANC1104
SANC1122
SANC1126
RMSD (nm)
B
Replicates of Holo Systems
SANC140
SANC382
SANC456
SANC522
SANC806
SANC968
SANC1082
SANC1095
SANC1104
SANC1122
SANC1126
RMSD (nm)
C
Replicates of Holo Systems
SANC382
SANC456
SANC522
SANC806
SANC1082
SANC1095
SANC1104
SANC1122
SANC1126
RMSD (nm)
Time (ns)
Holo_1
Holo_2
Holo_3
Pf,HsIleRS-SANC

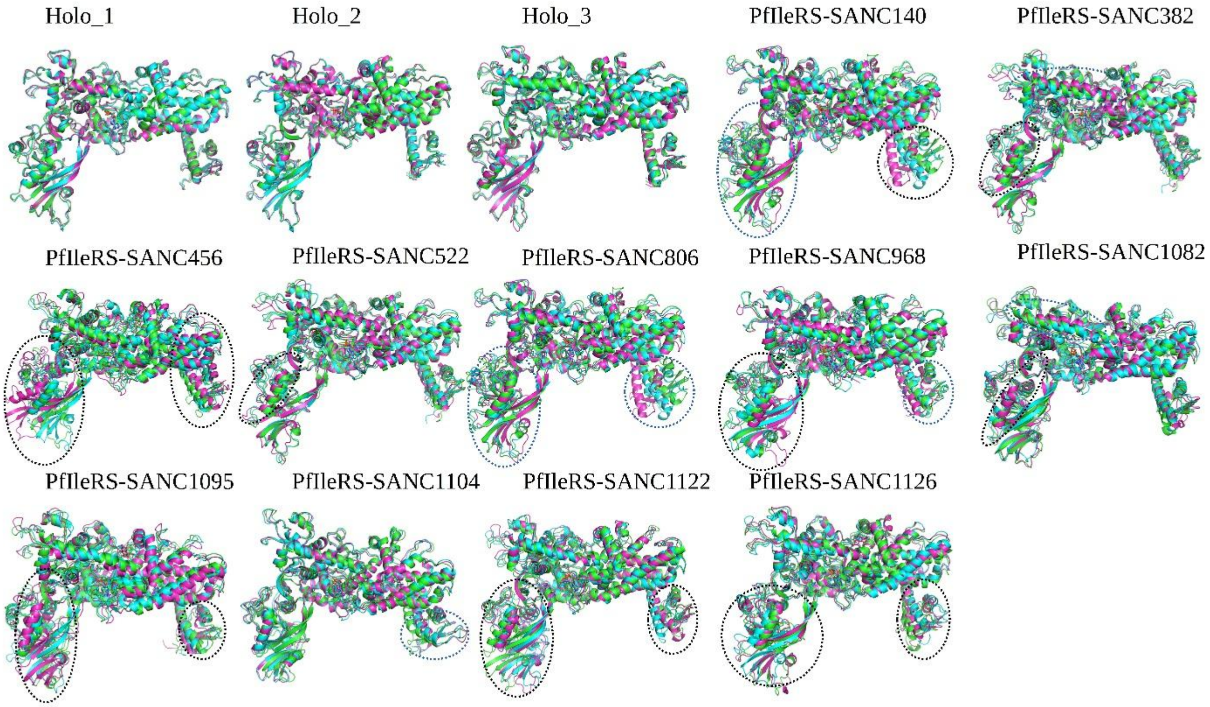
Holo_1
Holo_2
Holo_3
PfIleRS-SANC140
PfIleRS-SANC382
PfIleRS-SANC456
PfIleRS-SANC522
PfIleRS-SANC806
PfIleRS-SANC968
PfIleRS-SANC1082
PfIleRS-SANC1095
PfIleRS-SANC1104
PfIleRS-SANC1122
PfIleRS-SANC1126

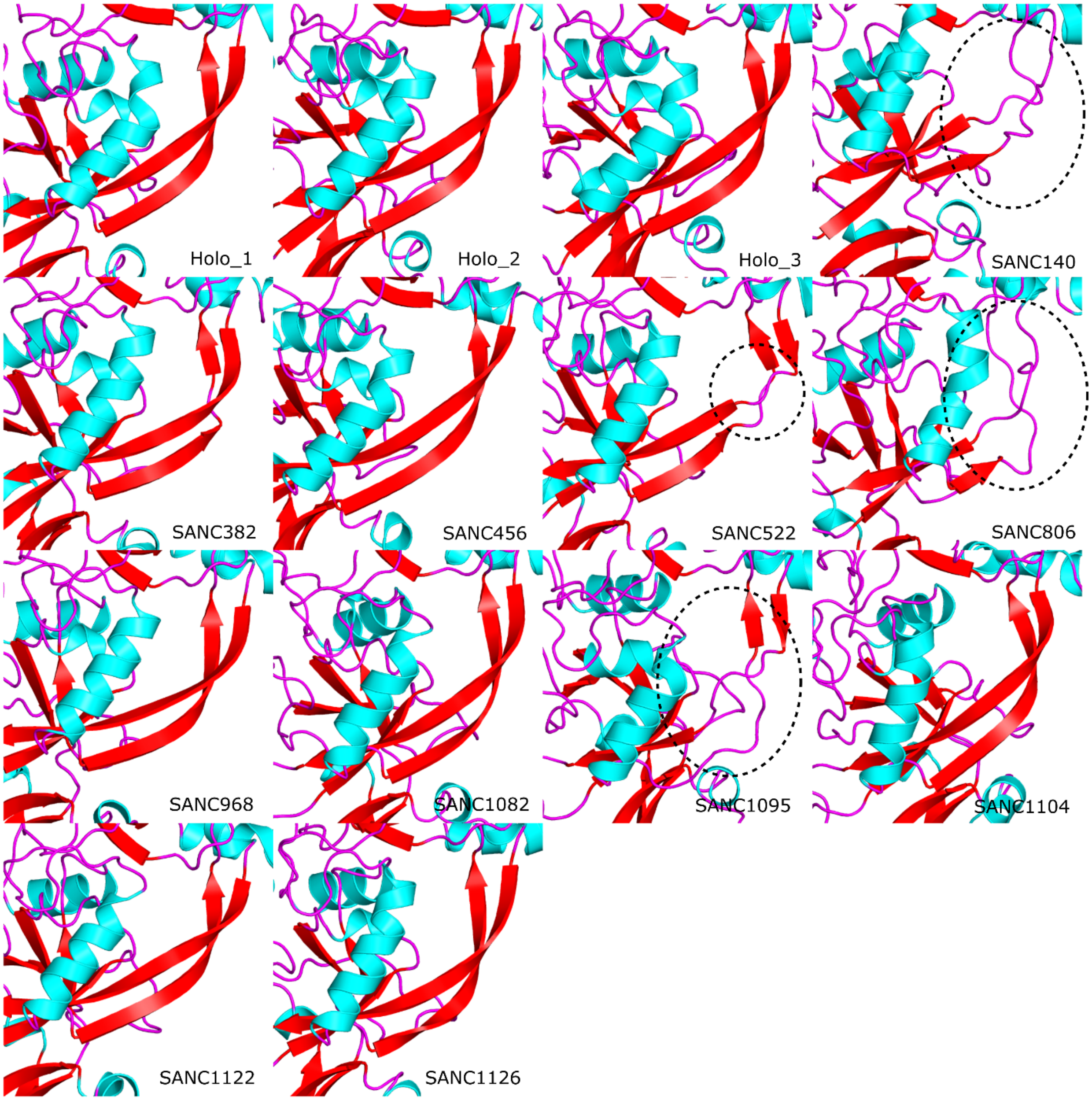
Holo_1
Holo_2
Holo_3
SANC140
SANC382
SANC456
SANC522
SANC806
SANC968
SANC1082
SANC1095
SANC1104
SANC1122
SANC1126

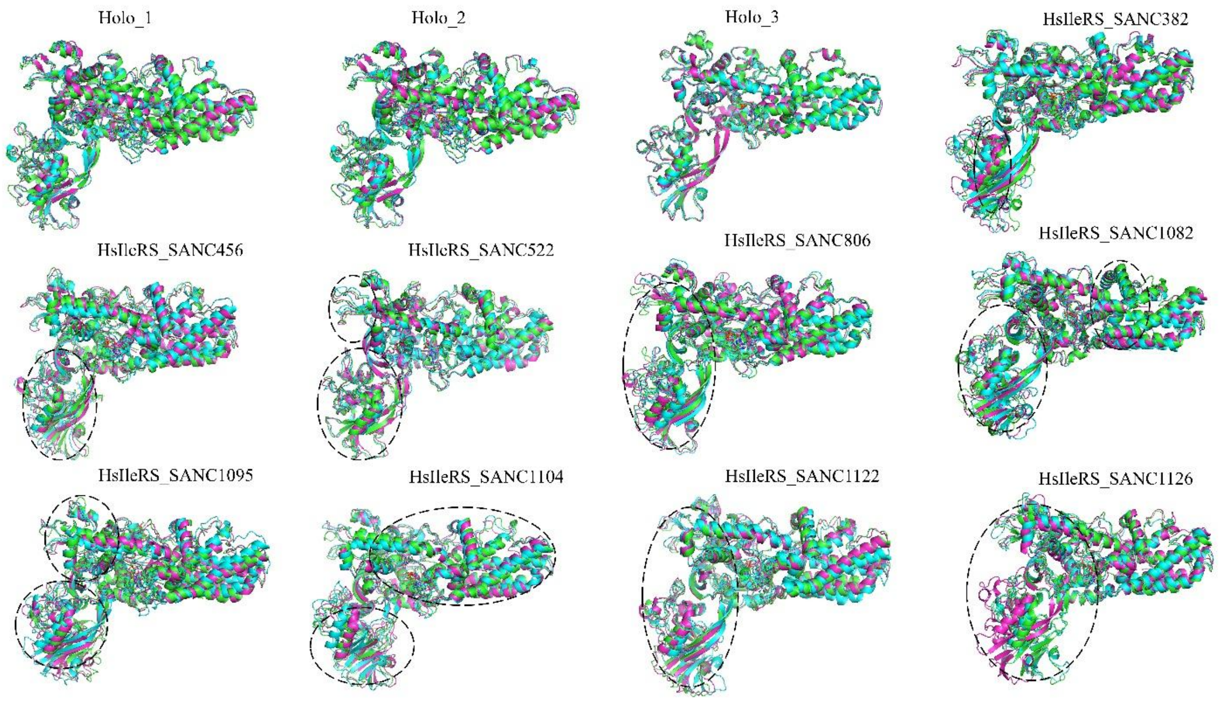
Holo_1
Holo_2
Holo_3
HsIleRS_SANC382
HsIleRS_SANC456
HsIleRS_SANC522
HsIleRS_SANC806
HsIleRS_SANC1082
HsIleRS_SANC1095
HsIleRS_SANC1104
HsIleRS_SANC1122
HsIleRS_SANC1126

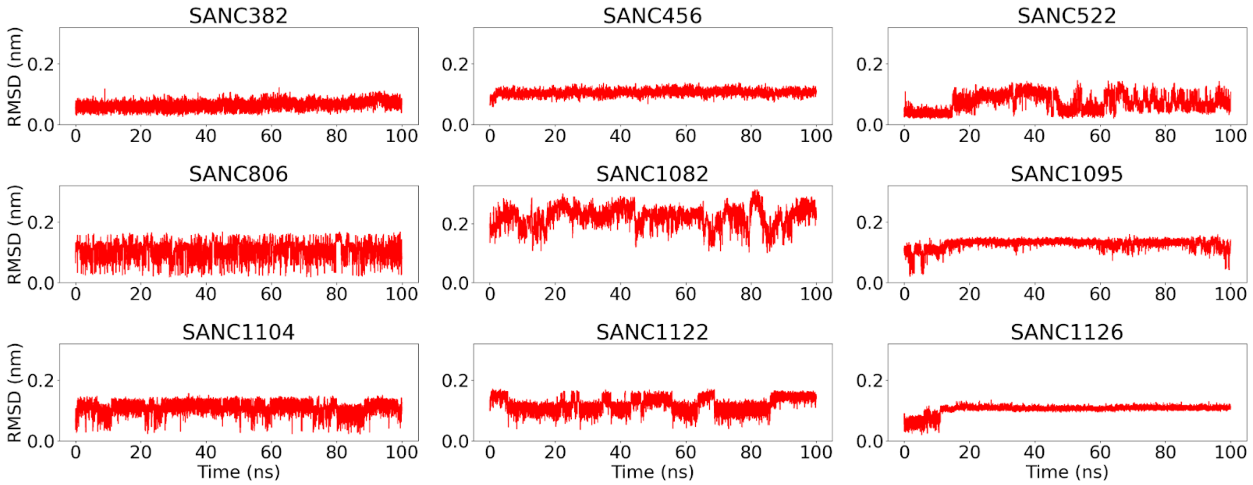
SANC382
SANC456
SANC522
SANC806
SANC1082
SANC1095
SANC1104
SANC1122
SANC1126
RMSD (nm)
0.0
0.2
0
20
40
60
80
100
Time (ns)

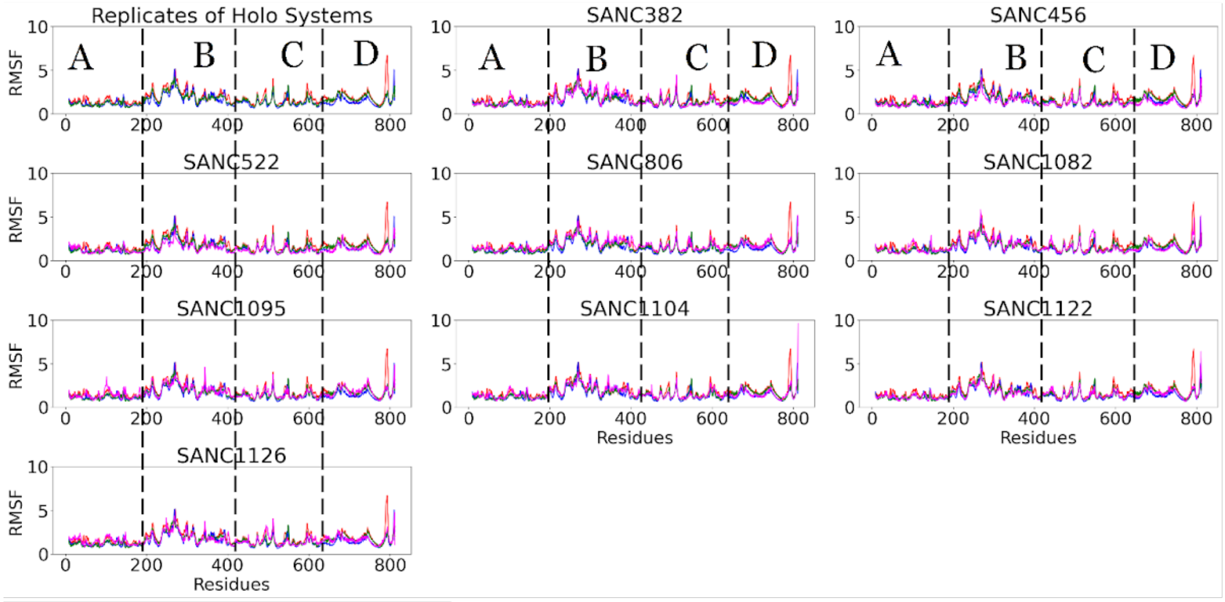

Simulation system	Colour code
Holo_1	
Holo_2	
Holo_3	
HsIleRS-SANC Complexes	

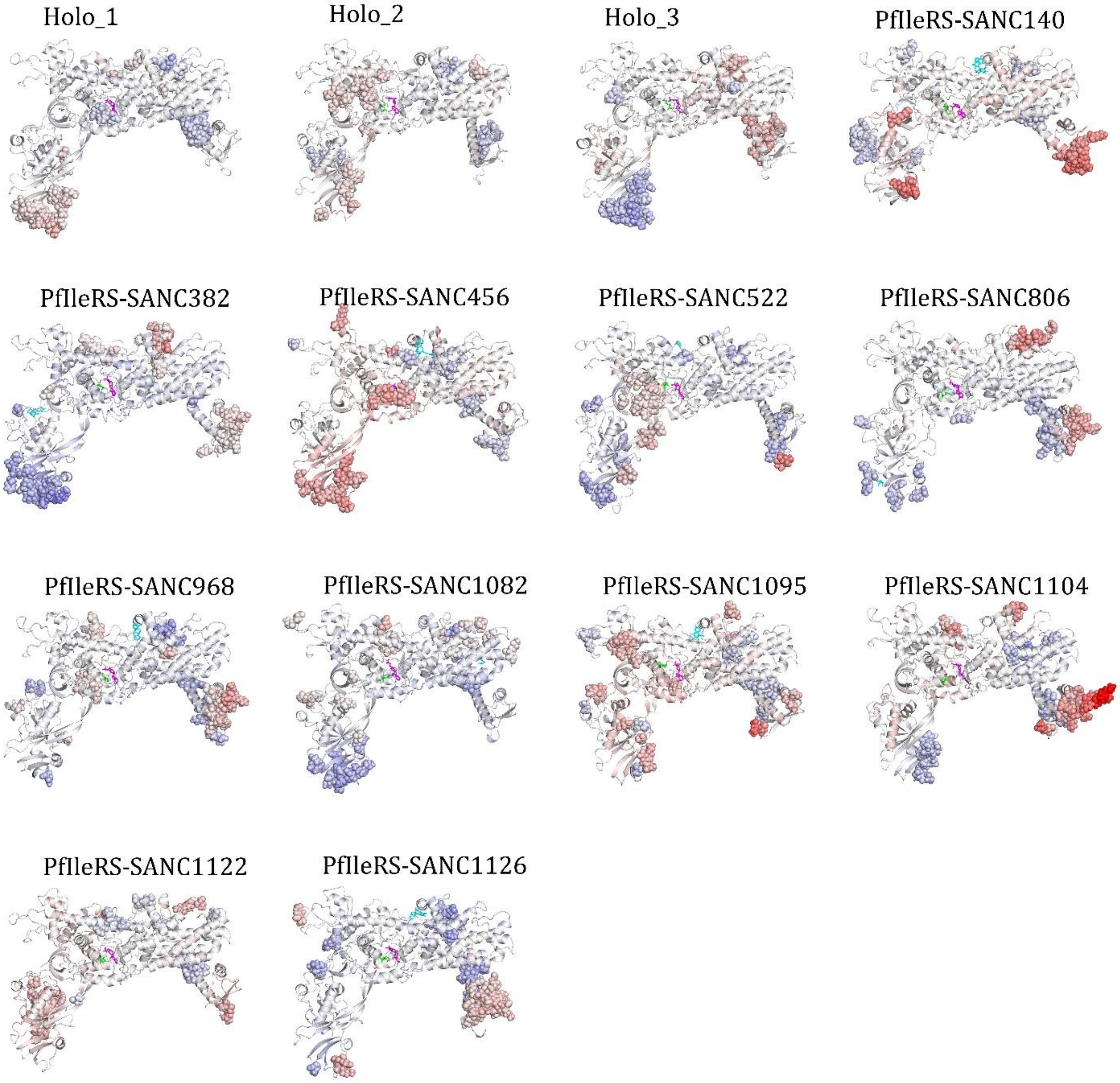
Holo_1
Holo_2
Holo_3
PfIleRS-SANC140
PfIleRS-SANC382
PfIleRS-SANC456
PfIleRS-SANC522
PfIleRS-SANC806
PfIleRS-SANC968
PfIleRS-SANC1082
PfIleRS-SANC1095
PfIleRS-SANC1104
PfIleRS-SANC1122
PfIleRS-SANC1126

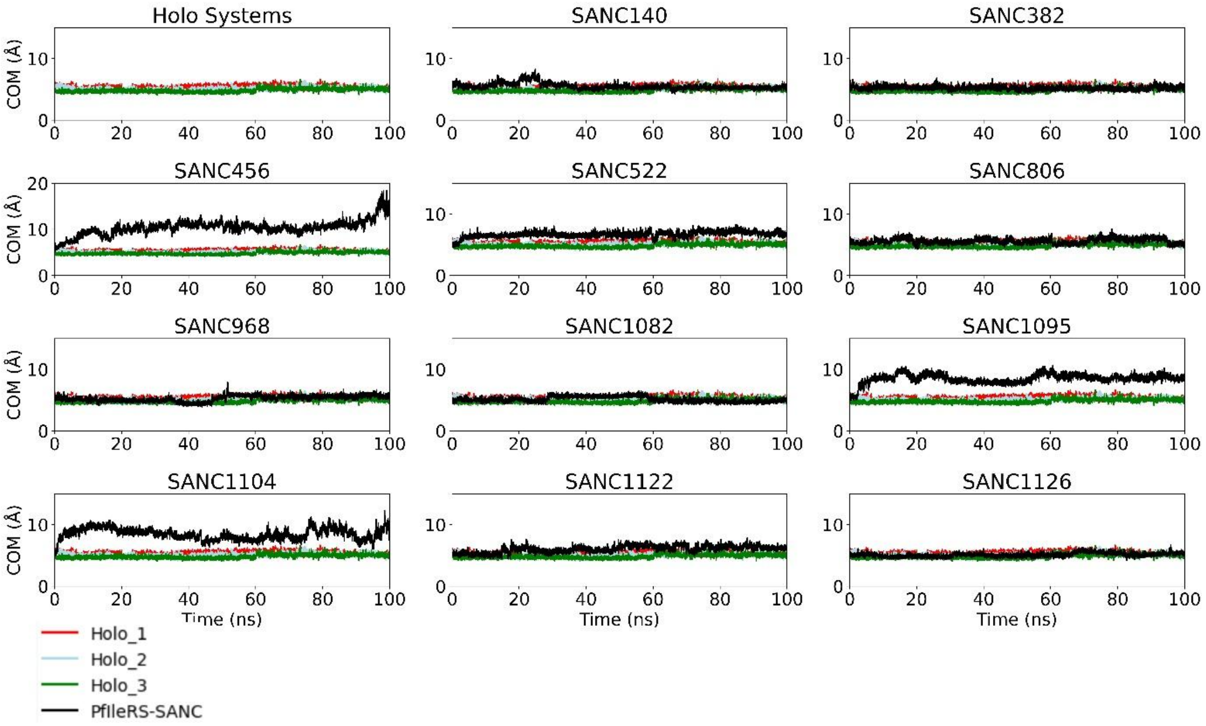
Holo Systems
SANC140
SANC382
SANC456
SANC522
SANC806
SANC968
SANC1082
SANC1095
SANC1104
SANC1122
SANC1126
COM (Å)
Time (ns)
Holo_1
Holo_2
Holo_3
PfIleRS-SANC

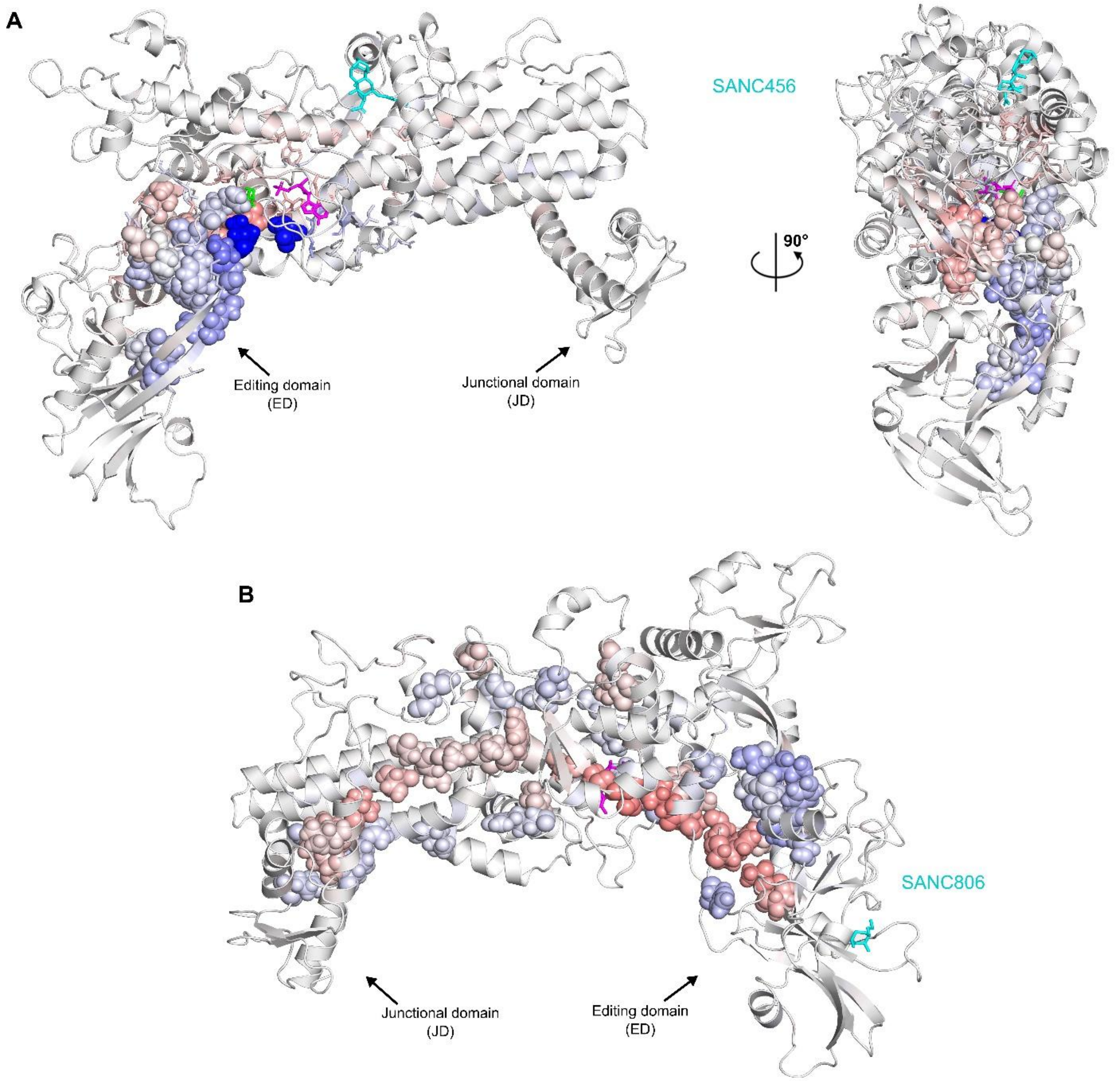
A
SANC456
90°
Editing domain
(ED)
Junctional domain
(JD)
B
SANC806
Junctional domain
(JD)
Editing domain
(ED)

Holo_1

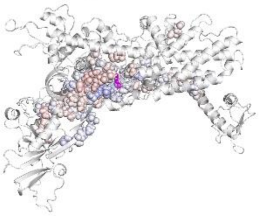

Holo_2

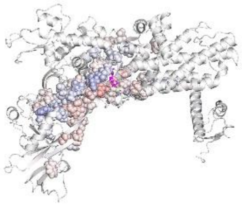

Holo_3

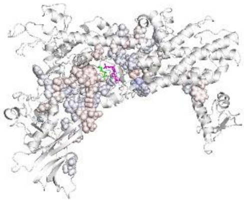

PfIleRS-SANC140

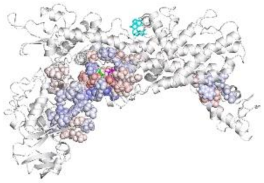

PfIleRS-SANC382

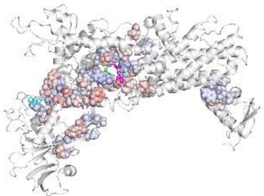

PfIleRS-SANC456

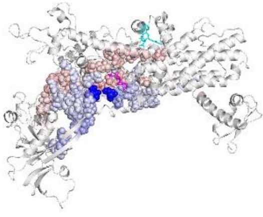

PfIleRS-SANC522

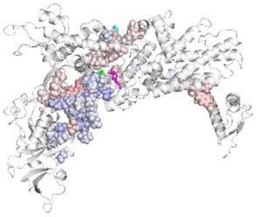

PfIleRS-SANC806

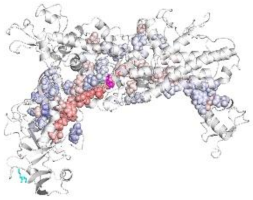

PfIleRS-SANC968

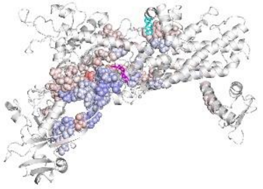

PfIleRS-SANC1082

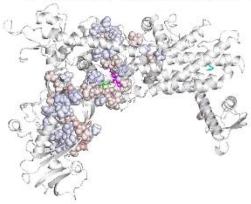

PfIleRS-SANC1095

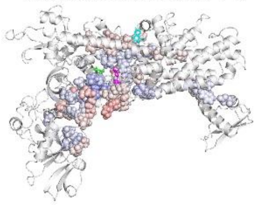

PfIleRS-SANC1104

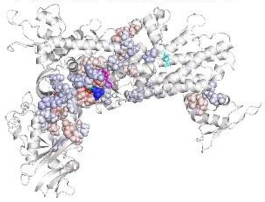

PfIleRS-SANC1122

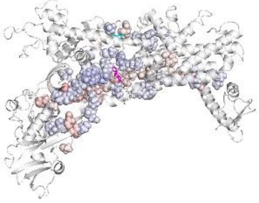

PfIleRS-SANC1126

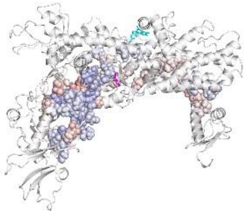

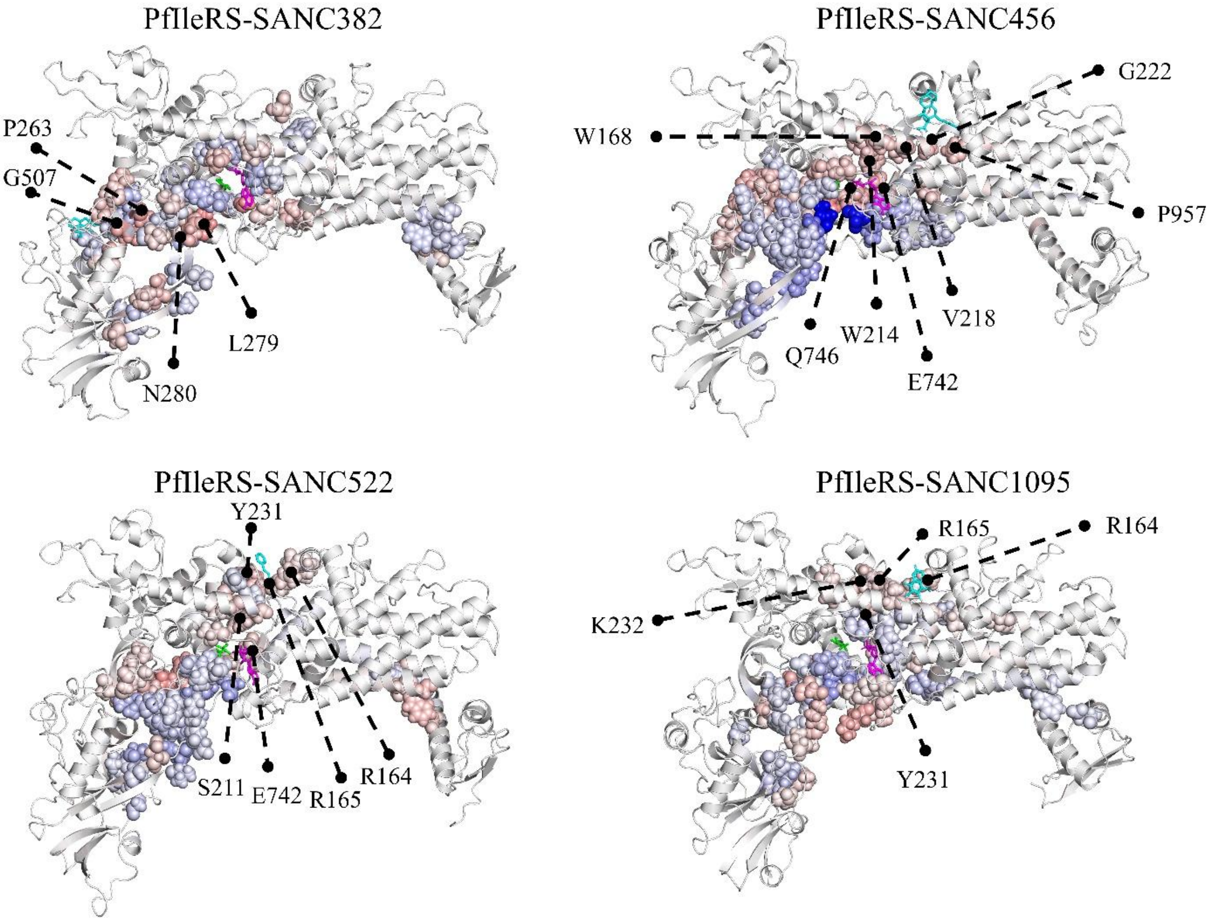
PfIleRS-SANC382
P263
G507
L279
N280
PfIleRS-SANC456
G222
W168
P957
Q746
W214
V218
E742
PfIleRS-SANC522
Y231
S211
E742
R165
R164
PfIleRS-SANC1095
R165
R164
K232
Y231

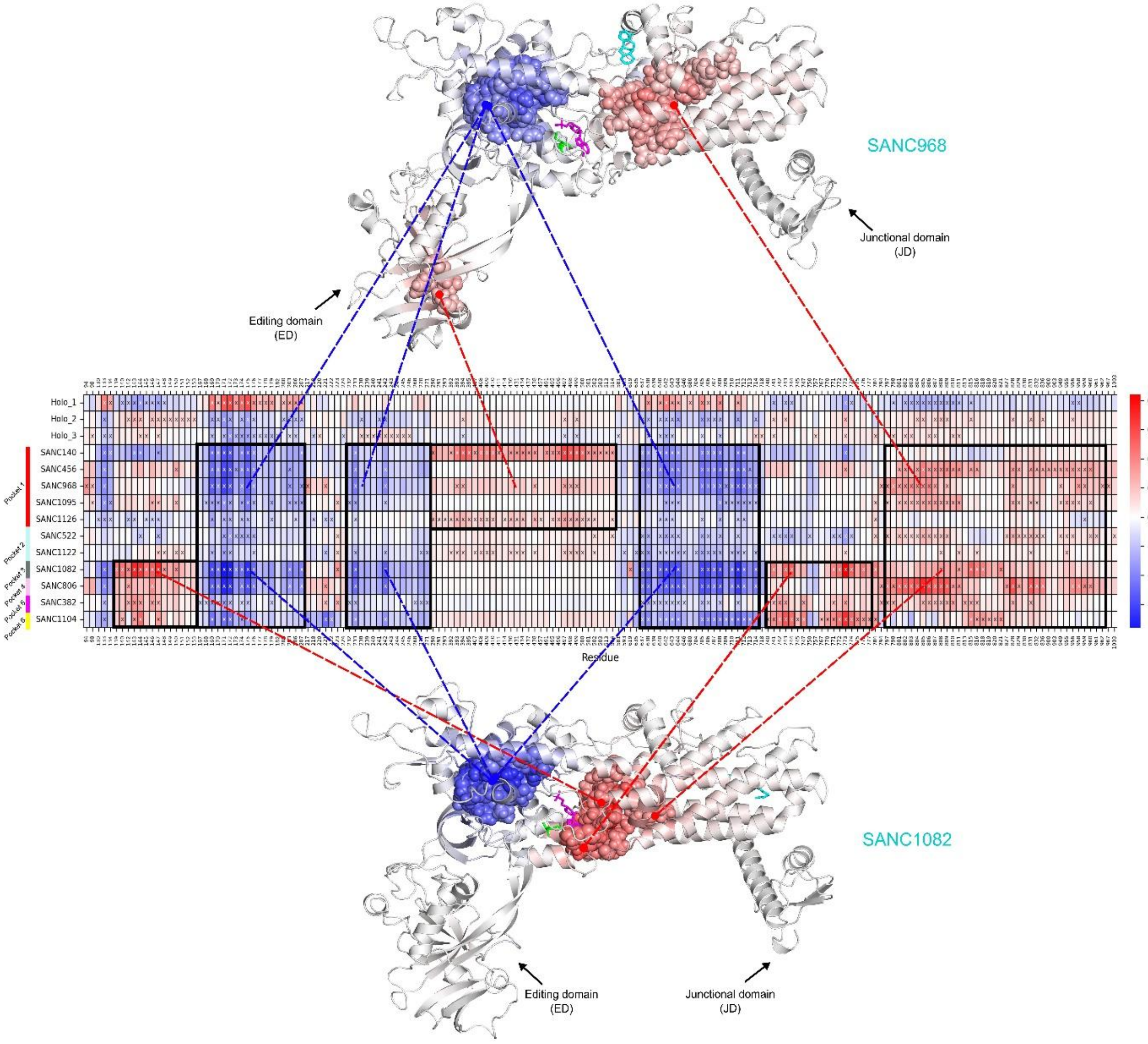
SANC968
Junctional domain
(JD)
Editing domain
(ED)
Holo_1
Holo_2
Holo_3
SANC140
SANC456
SANC968
SANC1095
SANC1126
SANC522
SANC1122
SANC1082
SANC806
SANC382
SANC1104
Pocket 1
Pocket 2
Pocket 3
Pocket 4
Pocket 5
Pocket 6
Residue
0.08
0.06
0.04
0.02
0.00
−0.02
−0.04
−0.06
SANC1082
Editing domain
(ED)
Junctional domain
(JD)

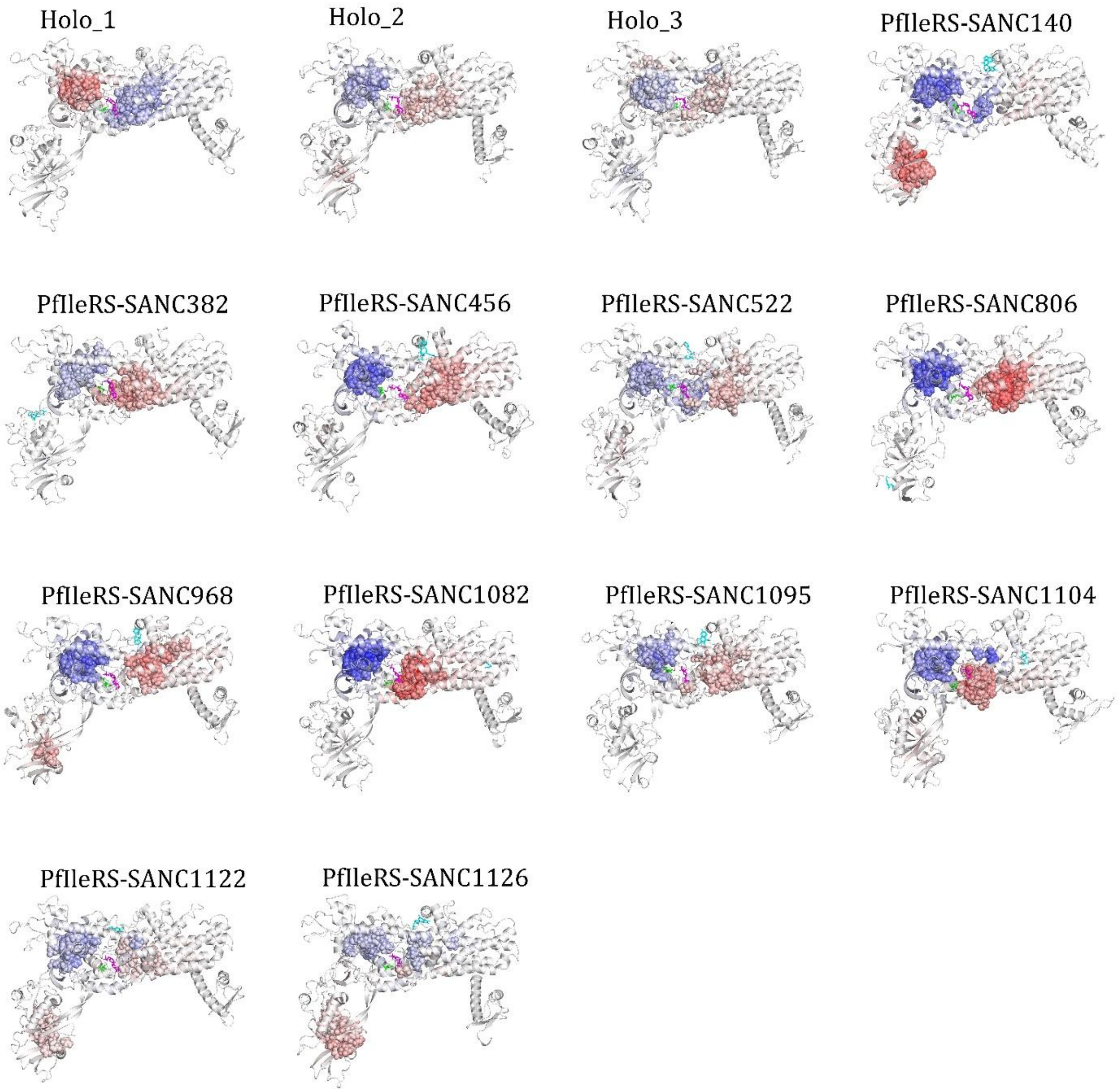
Holo_1
Holo_2
Holo_3
PfIleRS-SANC140
PfIleRS-SANC382
PfIleRS-SANC456
PfIleRS-SANC522
PfIleRS-SANC806
PfIleRS-SANC968
PfIleRS-SANC1082
PfIleRS-SANC1095
PfIleRS-SANC1104
PfIleRS-SANC1122
PfIleRS-SANC1126

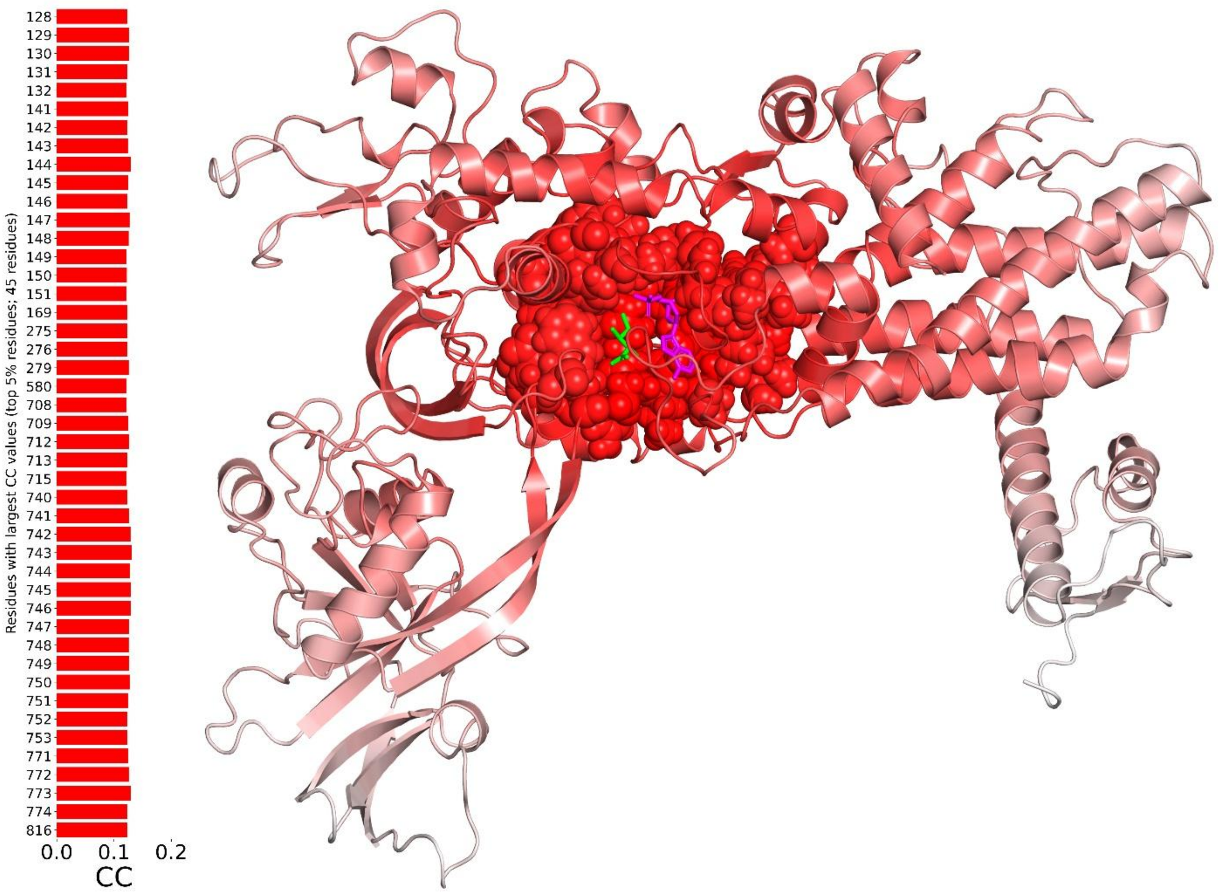
Residues with largest CC values (top 5% residues; 45 residues)
128
129
130
131
132
141
142
143
144
145
146
147
148
149
150
151
169
275
276
279
580
708
709
712
713
715
740
741
742
743
744
745
746
747
748
749
750
751
752
753
771
772
773
774
816
0.0
0.1
0.2
CC

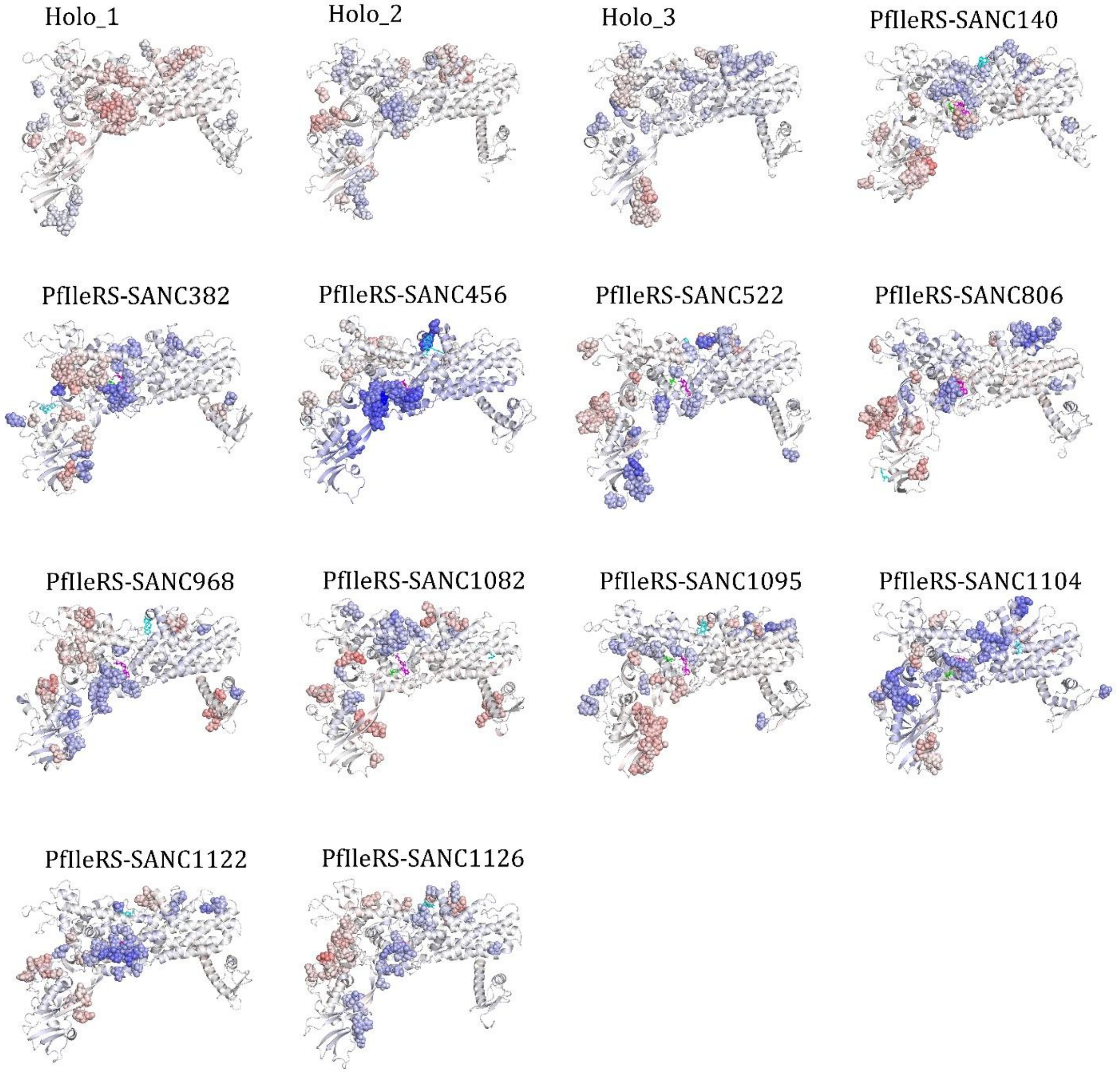
Holo_1
Holo_2
Holo_3
PfIleRS-SANC140
PfIleRS-SANC382
PfIleRS-SANC456
PfIleRS-SANC522
PfIleRS-SANC806
PfIleRS-SANC968
PfIleRS-SANC1082
PfIleRS-SANC1095
PfIleRS-SANC1104
PfIleRS-SANC1122
PfIleRS-SANC1126

Protein/ligands	MGLTools Scripts used	Box size	Box center
**PfIleRS**	*Prepare_receptor4.py*	110.60 Å x 87.12 Å x 114.61 Å	0.44 x, 7.24 y, and 4.54 z
**HsIleRS**	*Prepare_receptor4.py*	112.98 Å x 77.10 Å x 135.09 Å	-30.82 x, 24.24 y, and 9.15 z
**AMP**	*Prepare_ligand4.py*		

**Predicted allosteric site**	**SiteMap**	**DogSiteScorer**	**FTMap**	**PASSer**
**Pocket 1**	I101, L102, K103, W105, E106, D107, I108, D109, A110, F111, N112, L113, N115, R153, V215, V218, Q219, R220, G222, R223, W224, I225, D226, F227, K228, N229	L102, W105, E106, D109, A110, F111, N112, L113, V215, V218, Q219, G222, R223, W224, I225, D226, F227	L102, W105, E106, D109, A110, F111, N112, R153, V218, Q219, G222, R223, W224, I225	
**Pocket 2**	K119, K121, P123, Y124, I125, F126, Y127, D128, Y161, V162, E163, R164, R165, M715, P716, K719, V720, E727, D730, F731, I734	K121, P123, I125, D128, Y161, V162, R164, M715, P716, V720, E727, D730, F731, I734	P123, Y124, I125, F126, Y127, D128, Y161, V162, E163, R164, R165, M715, P716, K719, V720, E727, D730, F731, I734	

**Pocket 3**	F863, N864, T865, D866, Y867, I868, Y869, K870, N871, D872, N873, D876, Q877, F880, Q884, N939, V940, R943, Y946, L947, N977, M978, E979, H980, N981, D1012, K1014, Y1015, I1016, K1018	F863,T865,D866, Y867, Y869,K870,N871, D876, Q877,F880,Q884,N939,V940,R943,Y946, L947, N977,M978,E979, H980,N981,D1012, K1014, Y1015	F863,T865,V940,R943, Y946,L947,N977,M978,E979, H980	
**Pocket 4**	Y256, K257, S258, F259, K260, V261, P504, S505, Y511, I520, D521, P522, E523, S524, N525, I526, L527, I528, D529, A590, I591, P592, M654	Y256, F259, K260, V261, S524, N525, I526, L527, I528, D529, A590, I591, P592, M654	K260, V261, Y511, D521, P522, E523, S524, N525, I526, L527, I528, D529, A590, I591, M654	
**Pocket 5**	Y256, K260, V261, M262, P263, I272, S273, N274, L277, E508, Y511, R512, Y588, F595, S599, K632, D633, W634, C635	K260, V261, Y511, R512, Y588, F595, S599, K632, D633, W634, C635	Y256, K260, V261, M262, P263, I272, S273, N274, L277, E508, Y511, R512, Y588, F595, S599, K632, D633, W634, C635	
**Pocket 6**	N93, I94, E97, E98, I836, L837, Y840, I959, I963	N93, I94, E97, E98, I836, L837, Y840, I959, Y962, I963		N93, I94, E97, E98, I836, L837, Y840, I959, I963

**Pocket 7 (Editing domain)**	V261, L277, Y281, A394, W395, T396, T397, T398, W400, T401, L402, R434, F490, V491, S492, D493, D494, A495, G496, T497, G498, I499, V500, H501, C502, A503, Y506, G507, E508, D509, D510, Y511, H574, S575, Y576, P577, L586, I587, Y588, K632	V261, L277, Y281, A394, W395, T396, T397, T398, W400, T401, L402, R434, F490, V491, S492, D493, D494, A495, G496, T497, G498, I499, V500	L402, R434, F490, V491, S492, D493, D494, A495, G 496, T497, G498, I499, V500, H501, C502, A503, Y506, G507, E508, D509, D510, Y511, H574, S575, Y576, P577, L586, I587, Y588, K632	V261, L277, Y281, A394, W395, T396, T397, T398, W400, T401, L402, R434, F490, V491, S492, D493, D494, A495, G496, T497, G498, I499, V500, H501, C502, A503, Y506, G507, E508, D509, D510, Y511, H574, S575, Y576, P577, L586, I587, Y588, K632
**Pocket 8 (Orthosteric)**	D128, G129, P130, P131, F132, A133, T134, G140, H141, L142, K148, E213, W214, H279, K709, E742, G743, D745, Q746, R748, G749, W750, Q777, K783, M784, K786	D128, G129, P130, P131, F132, A133, T134, G140, H141, L142, K148, E213, W214, H279, K709, E742, G743, D745, Q746, R748, G749, W750, Q777, K783, M784, K786, L806	D128, G129, P130, P131, F132, A133, T134, G140, H141, L142, K148, E213, W214, H279, K709, E742, G743, D745, Q746, R748, G749, W750, Q777, K783, M784, K786, L806,	D128, G129, P130, P131, F132, A133, T134, G140, H141, L142, K148, E213, W214, H279, K709, E742, G743, D745, Q746, R748, G749, W750, Q777, L806, I836

Predicted allosteric site	SiteMap	DogSiteScorer	FTMap	PASSer
**Pocket 1**	I10,L11,E12,F13,W14[illegible] 5,E16,F17,N18,C19,F[illegible] Q21,E22,R62,F75,W1[illegible] K124,S125,V127,S12[illegible] 129,L130,G131,R132,[illegible] 33,I134,D135,F136,D[illegible] ,Y138	I10,L11,E12,F13,W14,T15,E16,F17,N18,C19,R62,F75,W123,K124,S125,V127,S128,R129,L130,G131,R132,W133,I134,D135,F136,D137,Y138		W14,T15,E16,F17,N18,C19,F20,Q21,E22,R62,F75,W123,K124,S125,V127,S128,R129,L130,G131,R132,W133,I134,D135,F136,D137,Y138
**Pocket 2**	Q21,L24,K32,F33,T34,F35,Y36,D37,H70,V71,D72,R73,R74,I134,D135,N138,D139,Y140,K141,Y144,Q146,F147,A528,Q529,V530,H531,E535,N536,E539,F540,A543	Q21,L24,K32,F33,T34,F35,Y36,D37,H70,V71,D72,R73,R74,I134,D135,N138,D139,Y140,K141,A528,Q529,V530,H531,E535,N536,E539,F540,A543	Q21,L24,K32,F33,T34,F35,Y36,D37,H70,V71,7D2,R73,R74,I134,D135,N138,D139,Y140,K141,Y144,Q146,F147,A528,Q529,V530,H531,E535	

**Pocket 3**	P31,F33,I56,I59,V60,T61,A64,H65,Q66,S67,G68,F69,D547,F548,K577,N578,I580,I694,G695,F697,E698,T699,M701,A702,A703,Y704,R705,L706,V709,P761,Y762,M7	P31,F33,I56,I59,V60,T61,A64,H65,Q66,S67,G68,F69,D547,F548,K577,I694,G695,F697,E698,T699,M701,A702,A703,Y762,M795,L796,P797,R798,V799		T61,A64,H65,Q66,S67,G68,F69,D547,F548,K577,N578,I580,I694,G695,F697,E698,T699,M701,A702,A703,Y704,R705L706,V709,P761,Y762,M795,L796

	95,L796,P797,R798, V799			
**Pocket 5**	Y165,V168,K169,V170,M171,P172,L181, S182,N183,F184,S186,H187,Y190,A318,P319,Y320,F321,G322,A323,E324,D325,Y326,M329,P340,Y401, A403,F408,N440,R445,D446,W447,T448	Y165,V168,K169, V170,M171,P172,L181,S182,N183,F184,S186,H187,Y190, A318,P319,Y320,F321,G322,A323,E324,D325,Y326,M329,P340,Y401,A403, F408,N440,R445,D446,W447,T448	Y165,V168,K169,V170,M171,P172,L181,S182,N183,F184,S186,H187,Y190,A318,P319,Y320,F321,G322,A323,E324,D325,Y326,M329,P340,Y401,A403,F408, N440,R445,D446,W447,T448	
**Pocket 7 (Editing domain)**	V170,M171,P172,Y190,A214,W215,T216, T217,T218,W220,T221,L222,S224,C229, R254,Y304,V305,K306,E307,E308,E309, G310,T311,V312,V313,H314,Q315,A316, P317,Y318,F320,G321,A322,E323,D324,F385,H387,S388,Y389,P390,L399,I400,Y401,K402,R445	V170,M171,P172, Y190,A214,W215, T216,T217,T218,R254,Y304,V305,K306,E307,E308,E309,G310,T311,V312, V313,H314,Q315, A316,P317,Y318,F320,G321,A322,E323,D324,F385,H387,S388,Y389,P390, L399	W215,T216,T217,T218,W220,T221,L222,S224,C229,R254,Y304,V305,K306,E307,E308,E309,G310,T311,V312,V313,H314,Q315,A316, P317,Y318,F320,G321, A322,E323,D324,F385, H387,S388,Y389,P390, L399,I400,Y401,K402, R445	

**Pocket 8 (Orthosteric)**	P40,F41,A42,T43,G44,H47,H50,P83,K90,W123,F184,E185,H187,Q188,N189,Y190,K191,C392,W393,R394,S395,D396,R436,W519,G552,I553,Q555,T556,R557,G558,W559,N582,G583,L584,Q591,K592,M593,S594,K595,S596,K597,E628	G44,H47,H50,P83,K90,W123,F184,E185,H187,Q188,N189,Y190,K191,R436,W519,G552,I553,Q555,T556,R557,G558,W559,N582,G583,L584,Q591,K592,M593,S594,K595,S596,K597,E628	H47,W123,F184,E185,H187,Q188,N189,Y190,K191,C392,W393,R394,3S95,D396,R436,W519,G552,I553,Q555,T556,R557,G558,W559,N582,G583,L584,Q591,K592,M593,S594,K595,S596,K597,E628	P40,F41,A42,T43,G44,H47,H50,P83,K90,W123,F184,E185,H187,Q188,N189,Y190,K191,C392,W393,R394,S395,D396,R436,W519,G552,I553,Q555,T556,R557,G558,W559,N582,G583,L584,Q591

Predicted pocket	Feature		PfIleRS	HsIleRS	Quantitative difference
**Pocket 1**	Conserved allosteric residues		K103, E106, D107, I108, D109, A110, N112, L113, N115, V215, Q219, K228	E12, F13, T15, E16, F17, N18, C19, Q21, E22, L24, K124, S127, D137	K103↔E12**;** positively to negatively charged E106↔T15; polar uncharged to negatively charged D109↔N18; negatively charged to hydrophobic V215↔K124; hydrophobic to positively charged K228↔137D; positively charged to negatively charged
	Pocket volume (Å)		153	193	40 (Å) smaller in PfIleRS
	Hydrophobic residues		I108	F13	I108↔F13; Increased hydrophobicity in Pf
**Pocket 2**	Conserved allosteric residues		Y124, I125, Y161, E163, K719, E727	F33, T34, H70, D72, Q194, N225	Y124↔F33; change in polarity Y161↔H70; hydrophobic to positively charged K719↔Q194; positively charged to polar uncharged
	Pocket volume (Å)		124	140	16 (Å) smaller in PfIleRS

	Hydrophobic residues		I125, Y161	F33	High hydrophobicity in PfIleRS
**Pocket 3**	Conserved residues		F863, T865, D866, Y867, I868, Y869, K870, F880, Y946, L947, K1015, K1018	Y672, E674, N675, T676, V709, R712, E735, L748, L752, S754, E768, D779	T865↔E674; polar uncharged to negatively charged I868↔V709; change in size Y869↔R712; hydrophobic to positively charged K870↔E735; positively charged to negatively charged
	Pocket volume				
	Hydrophobic residues		F863, Y867, I868,Y869, F880, Y946, L947	Y672, V709, L748, L752	Increased hydrophobicity in PfIleRS
**Pocket 5**	Conserved residues		L277, E508, S599, K632,V634	S256, A257, E323, R326,F329	L277↔S256; hydrophobic to polar uncharged E508↔A257; negatively charged to hydrophobic S599↔E323; polar uncharged to negatively charged
	Pocket volume (Å)		158	222	64 (Å) smaller in PfIleRS
	Hydrophobic		L277, V634	A257, F329	High hydrophobicity in

	residues				PfIleRS
**Pocket 7**	Conserved residues		S277, F490, S492, A495, I499, Y506, E508, K632	R197, S211, Y236, K306, V313, E309, F322, R410	**S277**↔R197; polar uncharged to positively A495↔K306; hydrophobic to positively charged I499↔V313; change in size
	Pocket size (Å)		208	214	6 (Å) smaller in PfIleRS
	Hydrophobic residues		F490, A495, I499, Y506	Y236, V313, F322	High hydrophobicity in PfIleRS

Active 2D structure	Bioactive ID	Predicted binding energy (Kac/mol)	Binding site
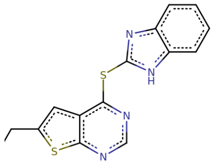	MMV019869	-9.7	Editing domain
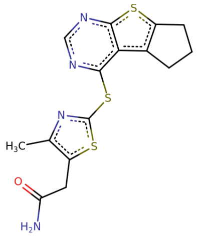	MMV1091186	-11.1	Active site
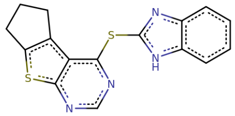	MMV007938	-8.7	Active site
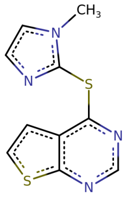	MMV062850	-9.9	Anticodon binding domain

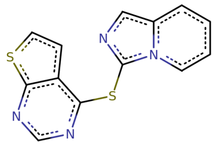	MMV019904	-10.3	Active site
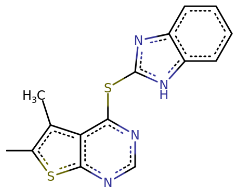	MMV019266	-8.1	Editing domain
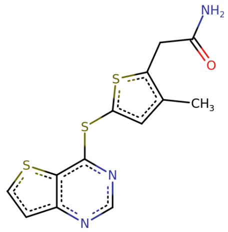	MMV1081413	-9.8	Editing domain
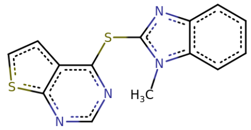	MMV019837	-8.1	Anticodon binding domain
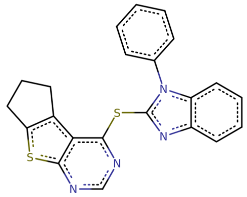	MMV1328428	-9.4	Active site

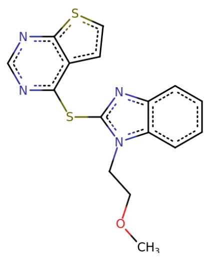	MMV020525	-7.8	Active site
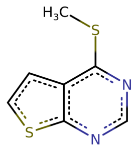	Mupirocin	–8.5	Editing domain

**Properties**	**SANC 140**	**SANC3 82**	**SANC4 56**	**SANC52 2**	**SANC806**	**SANC968**	**SANC108 2**	**SANC1095**	**SANC110 4**	**SANC1122**	**SANC1126**
Water solubility	✓	✓	✓	✓	✓	✓	✓	✓	✓	✓	✓
**Pharmacokinetics**											
Gastro-intestinal absorption (GI)	✓	✓	✓	✓	✓	✓	✓	✓	✓	✓	✓
**Druglikeness**											
Lipinski	✓	✓	✓	✓	✓	✓	✓	✓	✓	✓	✓
Ghose	✓	✓	✓	✓	✓	✓	✓	✓	✓	✓	✓
Veber	✓	✓	✓	✓	✓	✓	✓	✓	✓	✓	✓
Egan	✓	✓	✓	✓	✓	✓	✓	✓	✓	✓	✓
Muegge	✓	✓	✓	✓	✓	✓	✓	✓	✓	✓	✓
Bioavailability	0.55	0.55	0.55	0.55	0.55	0.55	0.55	0.55	0.55	0.55	0.55
**Medicinal Chemistry**											
PAINS	✓	✓	✓	✓	✓	✓	✓	✓	✓	✓	✓
Brenk	✓	✓	✓	✓	✓	✓	x	✓	x	✓	x
Leadlikeness	x	✓	x	✓	x	✓	x	✓	x	x	✓

✔ Pass, x Fail

Protein complex	Mean RMSD values
**Holo_1**	0.33
**Holo_2**	0.38
**Holo_3**	0.38
**PfIleRS-SANC140**	0.47
**PfIleRS-SANC382**	0.34
**PfIleRS-SANC456**	0.52
**PfIleRS-SANC522**	0.39
**PfIleRS-SANC806**	0.38
**PfIleRS-SANC968**	0.38
**PfIleRS-SANC1082**	0.28
**PfIleRS-SANC1095**	0.43
**PfIleRS-SANC1104**	0.34
**PfIleRS-SANC1122**	0.37
**PfIleRS-SANC1126**	0.39

**Protein complex**	**Mean RMSD values**
**Holo_1**	0.25
**Holo_2**	0.27
**Holo_3**	0.25
**HsIleRS-SANC382**	0.33
**HsIleRS-SANC456**	0.43
**HsIleRS-SANC522**	0.31
**HsIleRS-SANC806**	0.32
**HsIleRS-SANC1082**	0.25
**HsIleRS-SANC1095**	0.31
**HsIleRS-SANC1104**	0.33
**HsIleRS-SANC1122**	0.26
**HsIleRS-SANC1126**	0.29

Systems	Cluster 1	Cluster 2	Cluster 3
**Holo_1**	9423	314	263
**Holo_2**	9846	112	42
**Holo_3**	9952	34	14
**PfIleRS-SANC140**	7597	2325	78
**PfIleRS-SANC382**	8562	1377	61
**PfIleRS-SANC456**	5820	3845	335
**PfIleRS-SANC522**	9748	152	100
**PfIleRS-SANC806**	9575	1366	59
**PfIleRS-SANC968**	6453	3181	366
**PfIleRS-SANC1082**	9923	74	3
**PfIleRS-SANC1095**	6365	3629	6
**PfIleRS-SANC1104**	6488	3350	162
**PfIleRS-SANC1122**	8755	1164	81
**PfIleRS-SANC1126**	6898	3037	65

System	Cluster 1	Cluster 2	Cluster 3
**Holo_1**	9123	684	193
**Holo_2**	8998	832	170
**Holo_3**	9456	351	193
**HsIleRS-SANC382**	5592	3941	467
**HsIleRS-SANC456**	9613	361	26
**HsIleRS-SANC522**	5257	4469	274
**HsIleRS-SANC806**	5433	2656	1910
**HsIleRS-SANC1082**	5213	4194	593
**HsIleRS-SANC1095**	9673	221	106
**HsIleRS-SANC1104**	9471	527	2
**HsIleRS-SANC1122**	6038	3828	134
**HsIleRS-SANC1126**	6622	1852	1526

PfIleRS Complexes	ΔE^{vdW} (kcal/mol)	ΔE^{ele} (kcal/mol)	ΔE^{gb} (kcal/mol)	ΔE^{surf} (kcal/mol)	ΔG^{gas} (kcal/mol)	ΔG^{solv} (kcal/mol)	ΔG^{bind} (kcal/mol)
**Holo_1**	-36.8±0.06	-321.6±0.62	272.2±0.24	-5.7±0.02	-349.4±0.53	266.5±0.54	**-82.8±0.17**
**Holo_2**	-40.1±0.014	-254.7±0.58	253.5±0.33	-5.5±0.02	-294.7±0.51	208.1±0.37	**-86.7±0.17**
**Holo_3**	-40.5±0.013	-289.4±0.62	284.9±0.29	-5.8±0.01	-329.9±0.43	284.1±0.26	**-79.7±0.12**
**PfIleRS-SANC140**	-43.5±0.01	-263.7±0.31	270.1±0.54	-5.1±0.03	-307.1±0.79	264.9±0.58	**-42.2±0.15**
**PfIleRS-SANC382**	-42.3±0.02	-231.1±0.41	227.9±0.61	-5.6±0.02	-273.3±0.65	222.3±0.42	**-50.9±0.12**
**PfIleRS-SANC456**	-40.2±0.04	-227.7±0.52	237.1±0.48	-4.8±0.01	-267.9±0.55	232.2±0.51	**-35.7±0.13**
**PfIleRS-SANC522**	-36.5±0.04	-306.4±0.32	289.3±0.25	-5.4±0.02	-342.9±0.32	283.9±0.25	**-58.9±0.01**
**PfIleRS-SANC806**	-43.9±0.05	-259.5±0.4	251.4±0.32	-5.7±0.03	-303.4±0.39	245.6±0.32	**-57.7±0.12**
**PfIleRS-SANC968**	-35.1±0.04	-312.1±0.25	304.7±0.24	-5.3±0.02	-347.2±0.24	299.5±0.24	**-47.7±0.12**
**PfIleRS-SANC1082**	-37.3±0.08	-321.1±0.49	294.2±0.38	-5.4±0.02	-358.4±0.47	288.8±0.38	**-49.5±0.13**
**PfIleRS-SANC1095**	-34.6±0.06	-266.5±0.42	260.8±0.33	-5.0±0.02	-301.1±0.4	255.8±0.33	**-45.3±0.10**
**PfIleRS-SANC1104**	-43.5±0.05	-288.4±0.30	277.6±0.29	-5.6±0.02	-331.8±0.30	272.1±0.29	**-59.7±0.01**
**PfIleRS-SANC1122**	-40.4±0.05	-247.1±0.45	254.8±0.42	-5.2±0.02	-287.6±0.45	249.6±0.42	**-38.1±0.13**
**PfIleRS-SANC1126**	-42.5±0.05	-330.3±0.29	302.9±0.24	-5.9±0.02	-372.7±0.29	297.3±0.24	**-57.7±0.17**

ΔE^{vdW}=*van der Waals forces,* ΔE^{ele} =*electrostatics,* ΔE^{gb} =*polar solvation energy,* ΔE^{surf}=*non-polar contribution to solvation free energy,* ΔG^{gas}=*gas phase energy,* ΔG^{solv}=*solvation free energy and* ΔG^{bind}=*binding free energy*

**PfIleRS Complexes**	**ΔE^{vdW} (kcal/mol)**	**ΔE^{ele} (kcal/mol)**	**ΔE^{gb} (kcal/mol)**	**ΔE^{surf} (kcal/mol)**	**ΔG^{gas} (kcal/mol)**	**ΔG^{solv} (kcal/mol)**	**ΔG^{bind} (kcal/mol)**
**PfIleRS-SANC140**	-39.6±0.03	-18.5±0.06	30.6±0.05	-3.9±0.04	-58.1±-.06	26.8±0.05	**-31.2±0.03**
**PfIleRS-SANC382**	-34.9±0.04	-127.9±0.16	135.1±0.16	-4.2±0.04	-162.9±0.17	130.9±0.16	**-31.9±0.05**
**PfIleRS-SANC456**	-48.5±0.04	-12.6±0.07	31.5±0.07	-5.4±0.03	-61.1±0.08	26.1±0.07	**-34.9±0.04**
**PfIleRS-SANC522**	-33.2±0.03	-21.3±0.06	29.8±0.04	-4.3±0.02	-54.5±0.06	25.4±0.04	**-28.9±0.04**
**PfIleRS-SANC806**	-10.6±0.13	-71.4±0.05	74.6±0.05	-1.5±0.01	-82.1±0.06	73.2±0.05	**-8.9±0.01**
**PfIleRS-SANC968**	-34.4±0.03	-67.5±0.13	73.1±0.14	-3.7±0.03	-101.9±0.14	69.3±0.14	**-32.5±0.03**
**PfIleRS-SANC1082**	-26.6±0.03	-13.8±0.03	24.5±0.04	-3.9±0.06	-40.4±0.05	20.5±0.04	**-19.8±0.04**
**PfIleRS-SANC1095**	-26.0±0.04	-22.8±0.09	27.8±0.08	-3.1±0.03	-48.8±0.09	24.6±0.08	**-24.2±0.04**
**PfIleRS-SANC1104**	-25.9±0.02	-5.2±0.03	12.2±0.03	-3.4±0.02	-31.1±0.04	8.8±0.03	**-22.3±0.02**
**PfIleRS-SANC1122**	-23.9±0.06	-10.6±0.07	17.1±0.06	-2.9±0.05	-34.5±0.01	14.2±0.05	**-20.4±0.07**
**PfIleRS-SANC1126**	-27.5±0.06	-10.7±0.01	21.4±0.01	-3.5±0.07	-38.3±0.01	17.9±0.14	**-20.3±0.05**
