# Supplementary material for: Allosteric modulation of Plasmodium falciparum Isoleucyl tRNA synthetase by South African natural compounds
Source: PLoS One. 2025 May 14;20(5):e0321444. doi: 10.1371/journal.pone.0321444 (PMC12077802; doi:10.1371/journal.pone.0321444)
Supplement: Attachment — Submitted filename: SB_PONE-D-24-49827_ReviewerComment.pdf [file pone.0321444.s002.pdf]

Reviewer Comment

Manuscript: Allosteric Modulation of *Plasmodium falciparum* Isoleucyl tRNA Synthetase by South African Natural Compounds

General comment

I found this manuscript to be a timely and innovative contribution to antimalarial drug discovery, particularly in its approach to targeting *Plasmodium falciparum* Isoleucyl-tRNA Synthetase (PfIleRS) with allosteric inhibitors. I appreciate the methodological rigor demonstrated in the study, which integrates molecular docking, MD simulations, binding free energy calculations, and dynamic residue network analysis. The findings offer valuable mechanistic insights into how allosteric modulation disrupts catalytic function, with detailed residue-level analyses highlighting key players in enzymatic activity and communication pathways. I find the focus on species-specific inhibitors particularly compelling, as it underscores the potential to minimize off-target effects. Additionally, the novelty of targeting allosteric pockets enhances the therapeutic relevance of this work, making it a significant contribution to the field.

Below are few comments and areas for improvement to be considered by the authors.

- I noticed that the manuscript uses the abbreviation PfIleRS for *Plasmodium falciparum* Isoleucyl-tRNA Synthetase, while a referenced paper (ref no. 8) uses cIRS for the same enzyme. While PfIleRS provides species-specific clarity and aligns with common abbreviations for homologous enzymes (e.g., HsIleRS for humans), it deviates from the terminology used in prior literature. This discrepancy could cause confusion for readers familiar with cIRS. I suggest the authors either align with the established abbreviation or include a brief explanation for their choice of PfIleRS to ensure clarity and consistency in nomenclature.

- While the study emphasizes selectivity for *PfIleRS*, the exclusion of compounds based on their binding to the orthosteric sites of *HsIleRS* overlooks potential binding to *HsIleRS* allosteric pockets. This omission raises concerns about off-target effects and necessitates further investigation.

- For compounds like SANC140 and SANC968, which only docked to *PfIleRS*, could this be due to limitations in the docking protocol rather than true selectivity? Why were these compounds only docked to *PfIleRS*, and was their binding to *HsIleRS* allosteric pockets evaluated?

- I believe this study does not account for entropy in binding free energy calculations, nor does it explore longer-term dynamics through extended MD simulations. I think longer MD simulations or enhanced sampling techniques could capture slow conformational changes and give further comprehensive insights into allosteric pathways. These limitations should be acknowledged.

- I was looking forward to extensive or a decent length of discussion about the implications of these findings for resistance development and propose specific steps for compound optimization, but I could not see that in the conclusion. It would be great if the authors could include these.

Recommendation

Overall, this manuscript presents a compelling case for targeting *PfIleRS* with allosteric inhibitors and is a valuable contribution to antimalarial research. Addressing the few concerns outlined above will significantly strengthen the impact and reliability of the findings. I recommend **minor revisions** to address these points before publication.
